# Sewage sludge fertilization affects microbial community structure and its resistome in agricultural soils

**DOI:** 10.1038/s41598-024-71656-0

**Published:** 2024-09-09

**Authors:** Liliana Serwecińska, Arnoldo Font-Nájera, Dominik Strapagiel, Jakub Lach, Wojciech Tołoczko, Małgorzata Bołdak, Magdalena Urbaniak

**Affiliations:** 1 European Regional Centre for Ecohydrology of the Polish Academy of Sciences, Tylna 3, 90‑364 Lodz, Poland; 2 Present Address: Biobank Lab, Department of Oncobiology and Epigenetics, Faculty of Biology and Environmental Protection, University of Lodz, Pomorska 139, 90-235 Lodz, Poland; 3 Department of Physical Geography, Faculty of Geographical Sciences, University of Lodz, Narutowicza 88, 90-139 Lodz, Poland; 4 Department of Agriculture and Environmental Chemistry, University of Agriculture in Krakow, Mickiewicza 21, 31-120 Kraków, Poland; 5 UNESCO Chair on Ecohydrology and Applied Ecology, Faculty of Biology and Environmental Protection, University of Lodz, Banacha 12/16, 90‑237 Lodz, Poland

**Keywords:** Antibiotic resistance genes, Environmental threat, Soil bacterial community, Soil fertilization, Agroecology, Ecosystem ecology, Agroecology, Ecosystem ecology, Ecology, Microbiology, Antimicrobials, Microbial communities, Microbial genetics

## Abstract

Global sewage sludge production is rapidly increasing, and its safe disposal is becoming an increasingly serious issue. One of the main methods of municipal sewage sludge management is based on its agricultural use. The wastewater and sewage sludge contain numerous antibiotic resistance genes (ARGs), and its microbiome differs significantly from the soil microbial community. The aim of the study was to assess the changes occurring in the soil microbial community and resistome after the addition of sewage sludge from municipal wastewater treatment plant (WWTP) in central Poland, from which the sludge is used for fertilizing agricultural soils on a regular basis. This study used a high-throughput shotgun metagenomics approach to compare the microbial communities and ARGs present in two soils fertilized with sewage sludge. The two soils represented different land uses and different physicochemical and granulometric properties. Both soils were characterized by a similar taxonomic composition of the bacterial community, despite dissimilarities between soils properties. Five phyla predominated, viz. Planctomycetes, Actinobacteria, Proteobacteria, Chloroflexi and Firmicutes, and they were present in comparable proportions in both soils. Network analysis revealed that the application of sewage sludge resulted in substantial qualitative and quantitative changes in bacterial taxonomic profile, with most abundant phyla being considerably depleted and replaced by Proteobacteria and Spirochaetes. In addition, the ratio of oligotrophic to copiotrophic bacteria substantially decreased in both amended soils. Furthermore, fertilized soils demonstrated greater diversity and richness of ARGs compared to control soils. The increased abundance concerned mainly genes of resistance to antibiotics most commonly used in human and animal medicine. The level of heavy metals in sewage sludge was low and did not exceed the standards permitted in Poland for sludge used in agriculture, and their level in fertilized soils was still inconsiderable.

Agricultural soils are often degraded by intensive exploitation, resulting in soil erosion and deterioration^1^. One possible way to reverse these negative effects is by increasing the carbon, phosphorus and nitrogen content of the soil with organic amendments such as sewage sludge^2,3^. Such treatment has been recommended as a method of sludge utilization by the EU Sewage Sludge Directive 86/278/EEC (SSD) and the Urban Wastewater Treatment Directive 91/271/EC. Of all the sewage sludge generated in Europe during 2019, 50% was used in agriculture, 28% was incinerated, 18% was transported to landfills, and 4% used in other areas, such as land reclamation and forestry^4^. Due to the global rise in wastewater production and thus, wastewater sediment, effective sludge management and the long-term risk associated with its direct use in agriculture have become important issues of environmental research^5^. Municipal sewage sludge is formed as a by-product of many mechanical and biological wastewater treatment processes, and is treated with quicklime to make it safe for use, from a sanitary perspective. The resulting sludge is rich in easily-assimilable phosphorus and nitrogen compounds, as well as calcium that can deacidify soil; hence, it is suitable for use as a soil amendment to reduce the cost of agricultural production. Such use is currently permissible for the cultivation of cereals, for the reclamation of degraded areas, as well as cultivation of plants not intended for human and animal consumption^6^. Despite the benefits, there is the main concern that sludge can contain high levels of toxic substances, heavy metals, antibiotics, personal care products, pathogens and antibiotic-resistant bacteria (ARBs) from the inflowing wastewater; these contaminants can hence be introduced into agro-ecosystems through fertilization with sewage sludge^7–9^. Although the primary concern limiting the agricultural use of sewage sludge has been its heavy metal content, research over the last decade indicates that effluents and sewage sludge from WWTPs are significant sources of antibiotics and antibiotic resistance genes (ARGs) in the environment^10–12^. Although antibiotics have traditionally been used for medical and veterinary purposes, since the 1950s they have been added as growth promoters in poultry, livestock and in fish farming^10,13–15^. The resulting massive scale and overuse of antimicrobials has accelerated the evolution of ARBs and ARGs in the environment, thus indicating potential gene exchange between the environmental and clinical resistome^10,16,17^. ARBs have recently become emergent environmental pollutants and represent a potentially serious global threat to human and animal health, with a rise in MDR (multidrug-resistance) bacterial strains being documented worldwide^18–20^ The dramatic increase in the number of infections caused by drug-resistant bacteria worldwide has driven interest in tracking the environmental distribution of ARBs/ARGs and identifying their associated risk to public health^10^. The spread of ARBs and ARGs is exacerbated by the exchange of resistance genes between humans or animals and environmental microbiota. ARBs and ARGs are known to enter the environment via a range of pathways, although most studies are focused on farms with food-producing animals, the knowledge about the transmission routes for ARGs and ARBs in soil needs to be complemented. The presence of a mixture of antibiotics and large numbers of bacteria in wastewater, and in activated sewage sludge, encourages the transfer of ARGs, and thus the number of drug-resistant bacteria. For this reason, recent reports emphasize the need to expand the list of emerging contaminants present in sediments with ARB and ARG and to eliminate them from both sediments and fertilized soils^11,21,22^. Furthermore, the xenobiotics and resistant microorganisms present in wastewater sediments can shape the metabolic activity and diversity of the soil microbial community, and negatively affect the quality of the soil ecosystem^12^. Soil is the main source of microorganisms in terrestrial ecosystems, and its microbial communities are responsible for a number of metabolic processes that maintain soil welfare and ensure its fertility and quality. They play major roles in the mineralization of organic matter, nutrient availability, soil humus formation, and in limiting the number of pathogens. The land application of sewage sludge can influence the taxonomic structure of the indigenous soil microbial community; therefore, microbiome characteristics could be used as biological indicators to assess soil changes in response to sewage sludge amendment^21,23^. It was reported that some soil processes, such as nitrification, might be enhanced by fertilization with sewage sludge but at the same time other studies show different trends^22,24^. Some studies have demonstrated changes in the diversity and functional potential of soil microbial communities after agricultural use of dairy sewage sludge, straw, vermicompost or manures^25–27^; however, there are not many reports on the influence of the taxonomic structure of soil communities after treatment with municipal sewage sludge. Generally, our knowledge of how agricultural management shapes soil microbial community is still largely limited.

The aim of this study was to determine the short-term impacts of municipal sewage sludge application to farmland soil on the (1) soil bacterial community composition and diversity, and (2) the abundance and diversity of antimicrobial resistance genes. The study examined two different types of agricultural soils, i.e. sand and loamy sand, by a metagenomic approach.

## Results

### Soils characteristics

The soils used in the study were characterized by different granulometric and physicochemical properties (see Table 1). S1 was classified as sandy soil due to the high sand content of up to 85%, and S2 with a different composition as loamy sand (Table 1) according to the USDA and PSSS (Polish Society of Soil Science). Both types of soil are widespread in Poland. The pH value was another property that strongly distinguished both soils, 5.4 for S1 and 8.4 for S2. Moreover, both soils differed considerably in the content of *P* (0.05 in S1 and 0.16 cmol( +)·kg^−1^ in S2), Ca^2+^ (1.56 in S1 and 15.3 cmol( +)·kg^−1^ in S2), and Na^+^ (0.04 in S1 and 0.33 cmol( +)·kg^−1^ in S2). The greater the sum of alkaline cations in the soil and the higher pH value, the more nutrients for plants. This indicates greater availability of nutrients for plants in soil S2. The detailed granulometric composition and physicochemical parameters of both soils are depicted in Table 1, while Table S1 presents properties of sewage sludge.

**Table 1:** Granulometric and physicochemical properties of the soils used for the experiment.

Soil name	Granulometric composition	pH_KCl_	pH_H2O_	TOC [%]	Macroelements [g∙kg^−1^ of soil]					Cations exchange capacity				Sum of basic cations in soils
										[cmol( +)∙kg^−1^ of soil]				
					C_org_	N_org_	P	K	Mg	Ca^2+^	Mg^2+^	Na^+^	K^+^	
S1	85% sand	4.5	5.4	2.03	11.8	1.1	0.05	0.11	0.33	1.5	0.25	0.04	0.32	2.19
	9% silt													
	6% clay													
S2	62% sand	7.6	8.4	2.31	13.4	1.3	0.15	0.14	0.47	14.7	0.48	0.32	0.41	16.56
	24% silt													
	14% clay													

### Characteristics of bacterial population

**Fig. 1:**
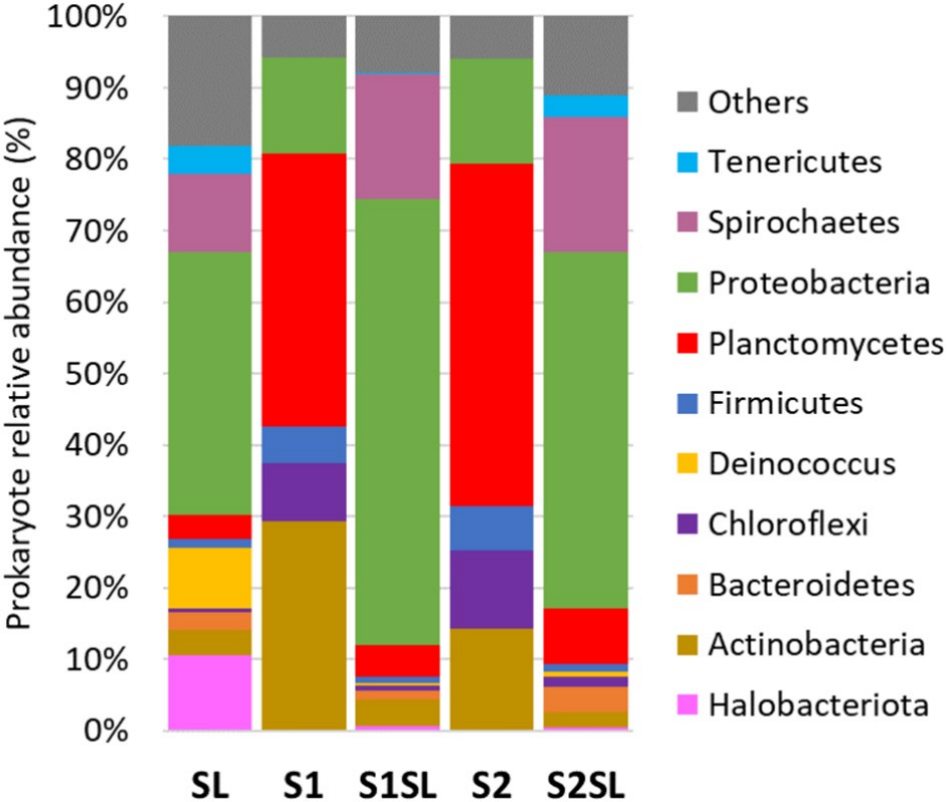
The relative abundance of prokaryotic phyla of the microbial community in applied sludge (SL), unamended soils (S1, S2) and soils amended with sludge (S1SL, S2SL). The graph describes only the taxonomic units that constituted > 1% of total relative abundance. All remaining taxa, i.e. those showing < 1% of total relative abundance are included in the category *others*.

Relative abundances (%) of prokaryotic communities at the phylum level were described in Fig. 1 and in the supplementary Table S2. Unfertilized soils S1 and S2 have very similar qualitative and quantitative taxonomic profiles at the phylum level. The principal bacterial members in these soils were found to belong to the Planctomycetes (38–48%), Actinobacteria (14–29%), Proteobacteria (13–15%), Chloroflexi (8–11%) and Firmicutes (5–6%) phyla (Fig. 1, Table S2). In turn, these five groups are poorly represented in the sewage sludge, used as a fertilizer (SL), however, sediment is dominated by Proteobacteria (37%), Spirochaetes (11%), Halobacteriota (10.5%) and Deinococcus (8.4%) (Fig. 1, Table S2). The fertilized soils S1SL and S2SL showed more similarities to SL, where the most dominant groups observed in the control soils have been considerably replaced by Proteobacteria and Spirochaetes (Fig. 1).

**Fig. 2:**
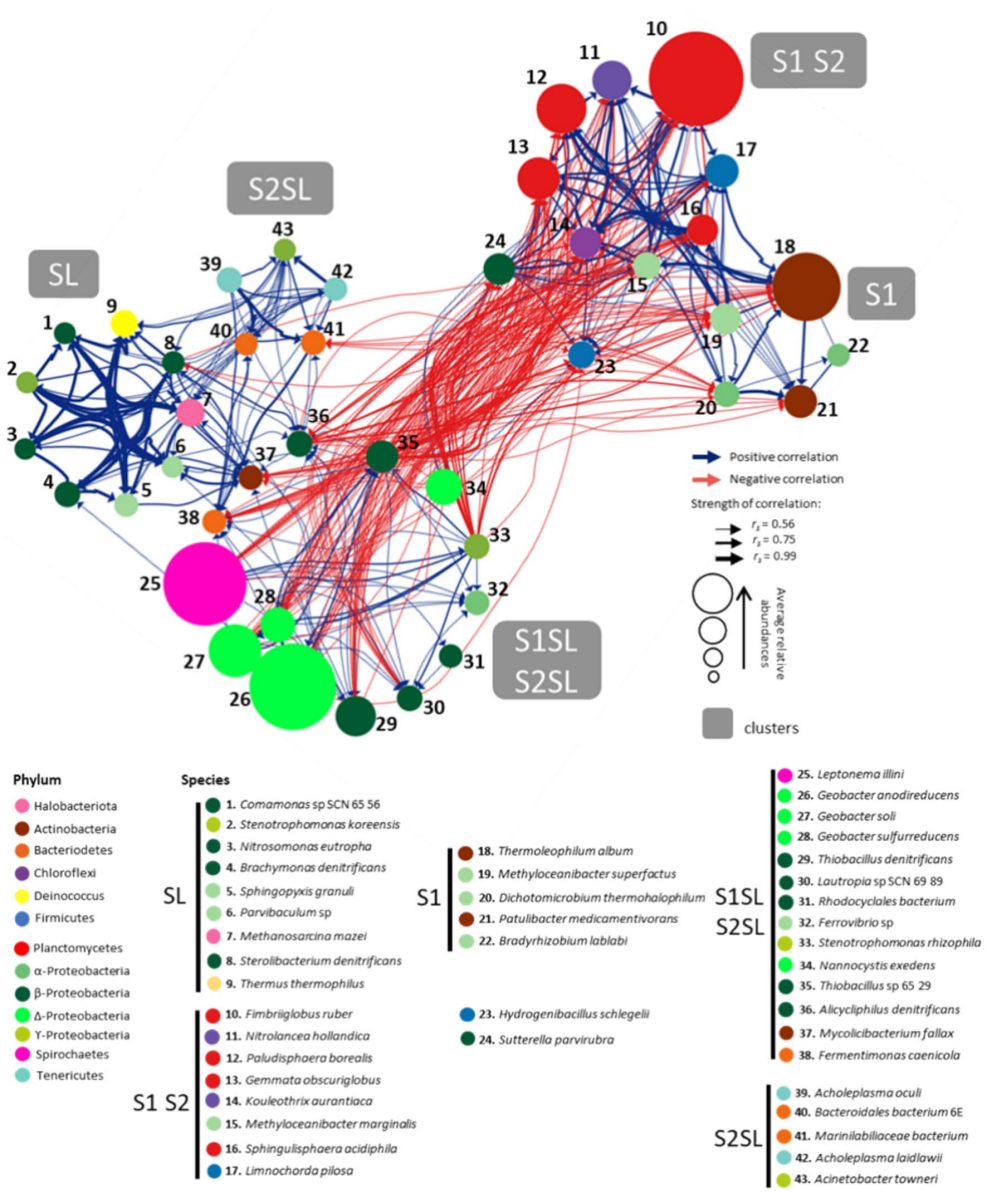
Co-occurrence network interactions between prokaryotes of the microbial community in sewage sludge (SL), unamended soil samples (S1, S2) and soils amended with sewage sludge (S1SL, S2SL). Interactions with significant Spearman’s correlations were considered for the analysis (*p* < 0.05 and *r*_*s*_ > 0.56). Node size is proportional to the average relative abundance and node color represents the phylum of the respective taxon. Edge color represents positive (blue) and negative (red) correlations between taxa, and the edge width represents the strength of the correlation. The network included only the taxa that contributed with at least 1% of the average total abundance. Cluster S1 S2 is composed of taxa with similar abundance between treatments S1 and S2, and cluster S1SL S2SL is composed of taxa with similar abundance between treatments S1SL and S2SL. Topological features for the construction of the network were detailed in supplementary Table S4 and overall correlations between clusters were summarized in Table S5.

The relationships between prokaryotic communities at different levels of treatment were investigated with co-occurrence network analysis using Spearman’s correlation (Fig. 2). Relative abundances (%) at the species level were also included in the network, however specific values were described in supplementary Table S3 and Fig. S1. Topological features for the construction of the network were described in supplementary Table S4 and Table S5. The network was constructed with a total of 365 significant correlations between taxa, from which positive relationships were more abundant than negative (222 over 143, respectively, Table S5). The network revealed two clusters composed of taxa mainly observed in the unfertilized soils (S1 S2 and S1), which were distanced from the other three clusters composed of taxa mainly observed for fertilized soils (S1SL S2SL and S2SL) and sewage sludge (SL) (Fig. 2). Such distance was supported by the highest number of negative relationships between the clusters from unfertilized soils when compared to the fertilized soils (138, Table S5), indicating that these treatments were significantly different from each other. In fact, species that were registered in large quantities for unfertilized soils, such as the Planctomycetes *Fimbriiglobus ruber* (21–27%), *Paludisphaera borealis* (9–10%), *Gemmata obscuriglobus* (5–7%) and *Sphingulisphaera acidiphila* (3–4%) and the Actinobacteria *Thermoleophilum album* (12–22%) and *Patulibacter medicamentivorans* (2–7%), were depleted in the fertilized soils (Figs. 2, S1, Table S3). Furthermore, almost no significant correlations were observed between clusters of unfertilized soils and SL (only 2 negative correlations, Table S5), suggesting that prokaryote composition between them is also different. In fact, many species that were registered with high abundance for SL, such as the β-Proteobacteria *Thiobacillus denitrificans* (9.1%) and *Brachymonas denitrificans* (7%), the α-Proteobacteria *Sphingopyxis granuli* (5.2%), the Deinococcus *Thermus thermophilus* (8.4%), and the Halobacteriota *Methanosarcina mazei* (10.5%), were not observed in unfertilized soils (Fig. 2, Table S3 and Fig. S1). In contrast, a higher number of positive correlations (34), when compared to negative (2), between fertilized soils (S1SL S2SL and S2SL) and sewage sludge (SL) clusters suggested a greater similarity between these treatments (Table S5). The high relative abundances of shared species between fertilized soils and SL, including the Spirochaetes *Leptonema illini* (11–19%) and β-Proteobacteria *T. denitrificans* (5–9%), corroborates the similarity between both treatments (Fig. 2, Table S3 and Fig. S1). An important element differentiating the fertilized soils was the composition of three species of ϒ-Proteobacteria belonging to the *Geobacter anodirudecens* (17–25%), *G. soli* (9–12%) *and G. sulfurreducens* (4–6%), which were generally depleted in any of the other treatments (Fig. 2, Table S3 and Fig. S1).

**Fig. 3:**
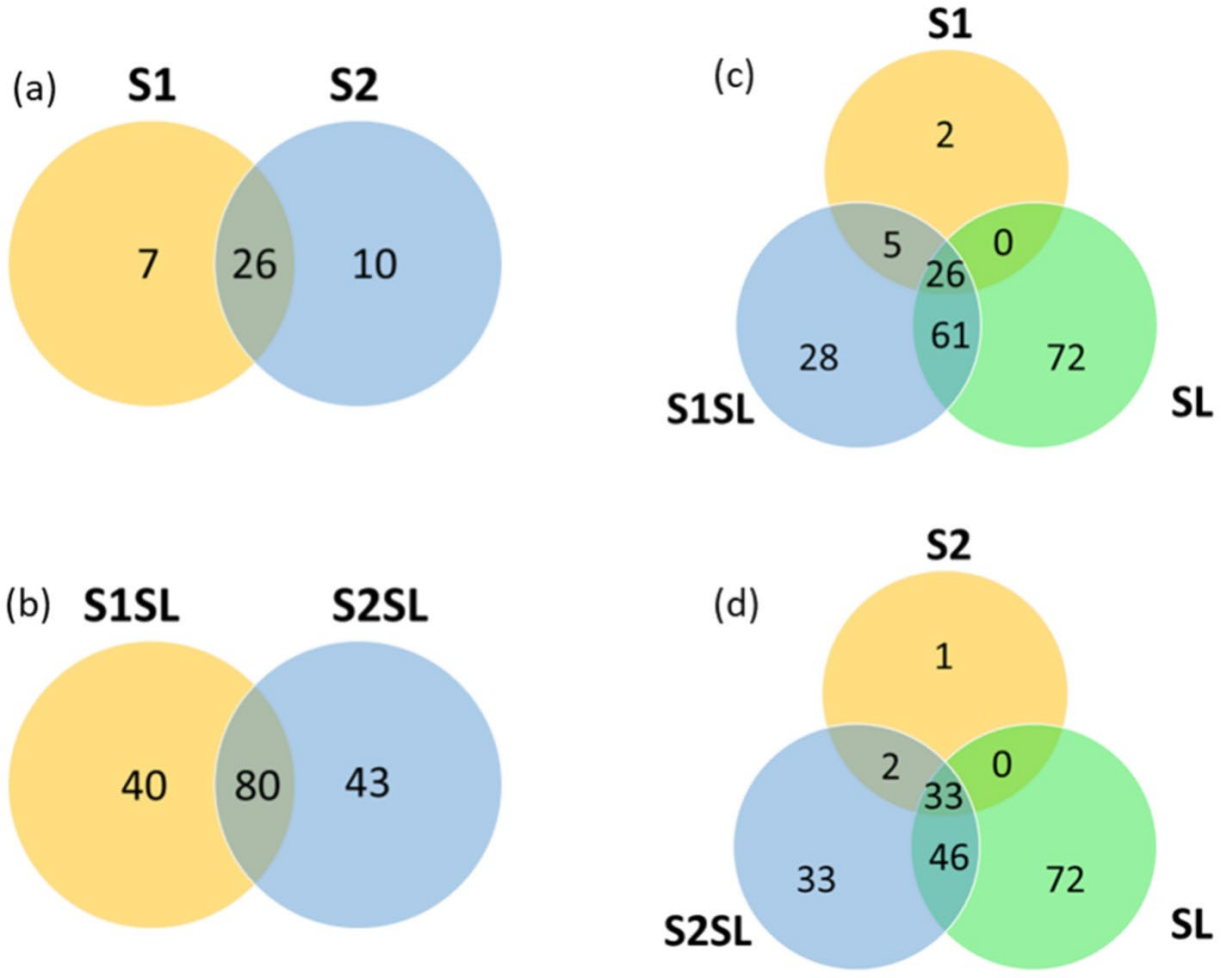
Venn diagram representing the distribution of the numbers of distinct and shared prokaryotic species in each sample. S1 and S2—unamended soils, S1SL and S2SL—soils amended with sewage sludge, SL—sludge. Estimation of values was performed with the total richness of taxa registered for the different samples.

The number of shared and distinct species between the different types of soils was summarized in the form of Venn diagrams in Fig. 3. A total of twenty six shared prokaryotic taxa were present in both unfertilized S1 and S2 soils (Fig. 3a). The number of shared species increased to eighty between fertilized S1SL and S2SL, and the number of distinct species increased to forty and forty three, respectively (Fig. 3b). In addition, only five species were shared and thirty were distinct between soil S1 and fertilized S1SL, and merely two were shared, and thirty four were exclusive between S2 and S2SL (Fig. 3c,d respectively). In contrast, sixty one taxa were shared for SL and S1SL and forty six for SL and S2SL. However, no prokaryotic species were shared between the sewage sludge and any of the control soils S1 and S2 (Fig. 3c,d).

**Fig. 4:**
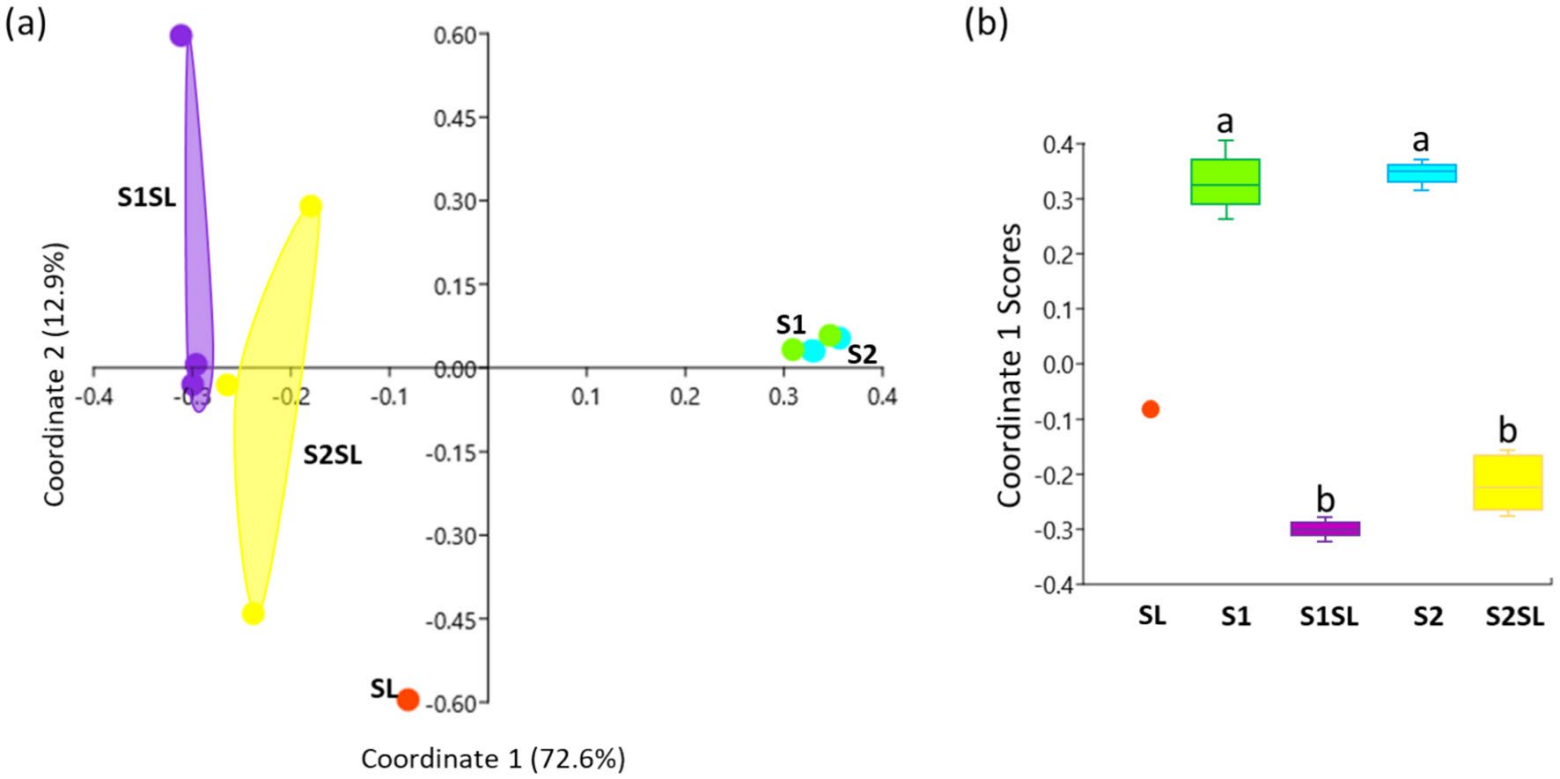
Principal coordinate analysis (PCoA) describing the effects of experimental treatments on the taxonomic composition of soil prokaryotic communities. Coordinates 1 and 2 explain the highest variability for all experiments (85.5%) (**a**), see supplementary Table S6. Statistically significant differences between samples using non-parametric Kruskal–Wallis and Mann–Whitney tests (*p < 0.05) (**b**), see supplementary Table S7. S1 and S2—unamended soils; S1SL and S2SL—soils amended with sewage sludge, SL—sewage sludge.

The differences in soil prokaryotic communities between all samples were described with principal coordinate analysis (PCoA) in Fig. 4. The horizontal axis (coordinate 1–72.6% of variability) indicates no differences in prokaryotic communities between the unfertilized S1 and S2 soils. These soils showed greater differences when compared to the fertilized soils (S1SL and S2SL) and to the sludge sample (Fig. 4a). The vertical axis (coordinate 2–12.9% of variability) indicates variation within replicate samples for the amended soils S1SL and S2SL (Fig. 4a). The two soils demonstrated greater differences in taxa between unfertilized controls and fertilized samples (horizontal axis), than between replicates (vertical axis) (Fig. 4a) which was also observed to be statistically significant (Fig. 4b).

**Fig. 5:**
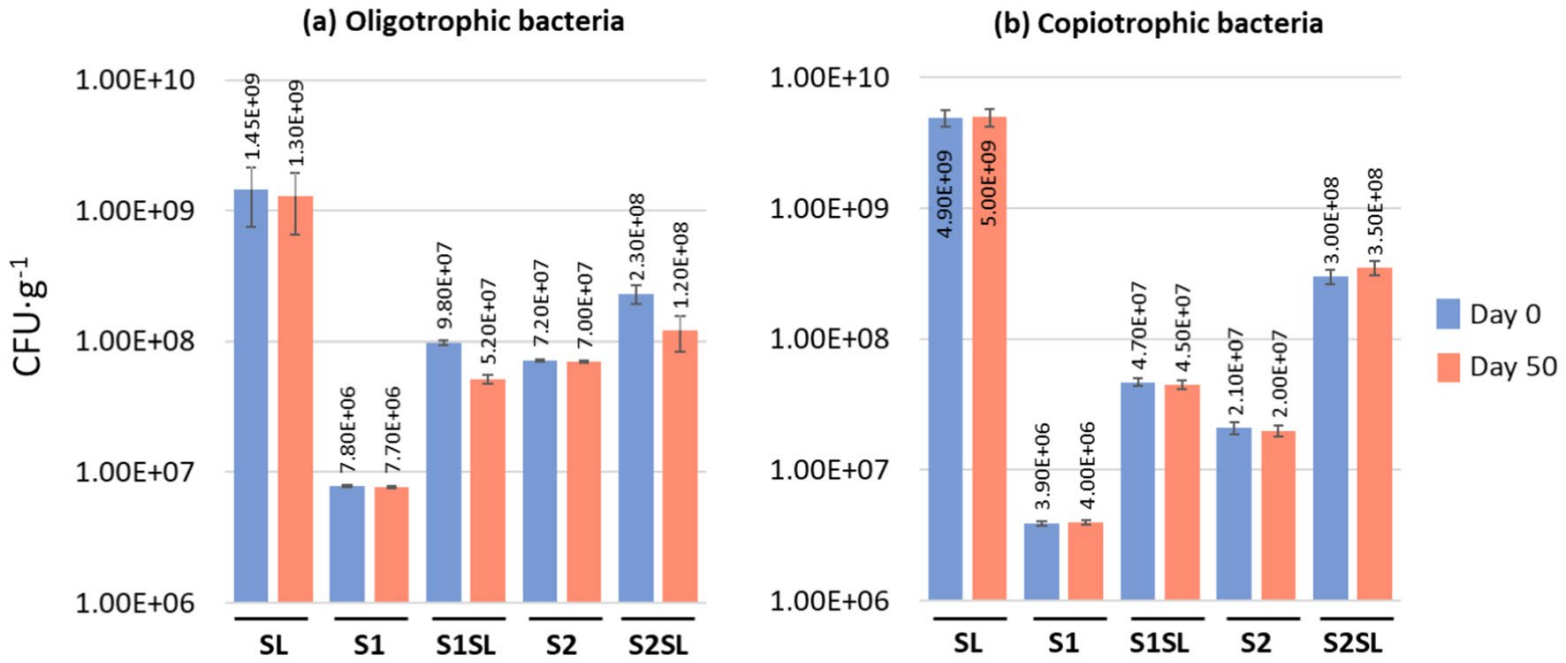
Variation of the abundance of oligotrophic and copiotrophic bacteria in the tested soils and sewage sludge. Results presented as colony forming units (CFU) per one gram of dry soil or sewage sludge. Numerical values of CFU∙g^−1^ were also presented on the X-axis.

The average abundance of oligotrophic and copiotrophic bacteria per gram of soil or sediment was presented in Fig. 5. The number of both, oligotrophs and copiotrophs in S2 was an order of magnitude higher compared to S1 (Fig. 5a, b), the S2 soil presented over three times more oligotrophs than copiotrophs, and in S1 the numerical ratio was 2:1 (Fig. 5a,b, Table S8). Moreover, the numbers of both group of bacteria and proportion between them in S1 and S2 did not change after seven weeks of experiment (Fig. 5a,b). The number of oligotrophs and copiotrophs in both soils increased by about one order of magnitude after fertilization in day 0, however, seven weeks later the abundance of oligotrophs decreased approximately twofold, whereas the abundance of copiotrophs remained at the same level in both, S1SL and S2SL. Thus, seven weeks after the application of sediment, the numerical ratio of oligotrophs to copiotrophs in both fertilized soils decreased considerably, i.e. approximately twice in S1SL compared to the S1 and as much as tenfold in S2SL compared to the S2 at day 0 (Table S8).

### The content of heavy metals and ARGs

The metal content in soils and sludge samples were summarized in Table 2. The very low level of metals was found in all tested soil samples, in some cases at the detection limit. Sewage sludge contained the highest quantity of all analyzed metals. All four experimental soils, both controls and fertilized samples, contained considerably lower levels of metals than SL (Table 2).

**Table 2:** Content of heavy metals in the tested samples (mg·kg^−1^ dry weight).

Metal	SL	S1	S1SL	S2	S2SL
Zn	629.87 ± 10.95	10.36 ± 3.73	19.19 ± 0.18	8.87 ± 4.17	9.04 ± 0.81
Cu	121.54 ± 7.02	2.74 ± 0.40	4.57 ± 0.45	3.87 ± 0.35	5.17 ± 1.73
Cr	30.49 ± 0.83	6.58 ± 0.29	6.83 ± 0.16	7.07 ± 0.37	7.24 ± 0.59
Pb	14.13 ± 0.87	8.61 ± 1.16	7.31 ± 1.20	7.51 ± 3.44	6.10 ± 1.50
Ni	13.56 ± 0.56	3.44 ± 0.17	3.36 ± 0.16	4.71 ± 0.34	4.57 ± 0.13
Fe	6.31 ± 0.08	2.62 ± 0.07	2.55 ± 0.13	3.46 ± 0.15	3.57 ± 0.14
Mo	4.14 ± 0.06	0.21 ± 0.04	0.25 ± 0.02	ND	0.01 ± 0.01
Cd	1.32 ± 0.05	0.19 ± 0.06	0.07 ± 0.01	0.20 ± 0.03	0.13 ± 0.01

Standard deviation is shown for each media (n = 3). ND: not detected (below the limit of detection).

**Fig. 6:**
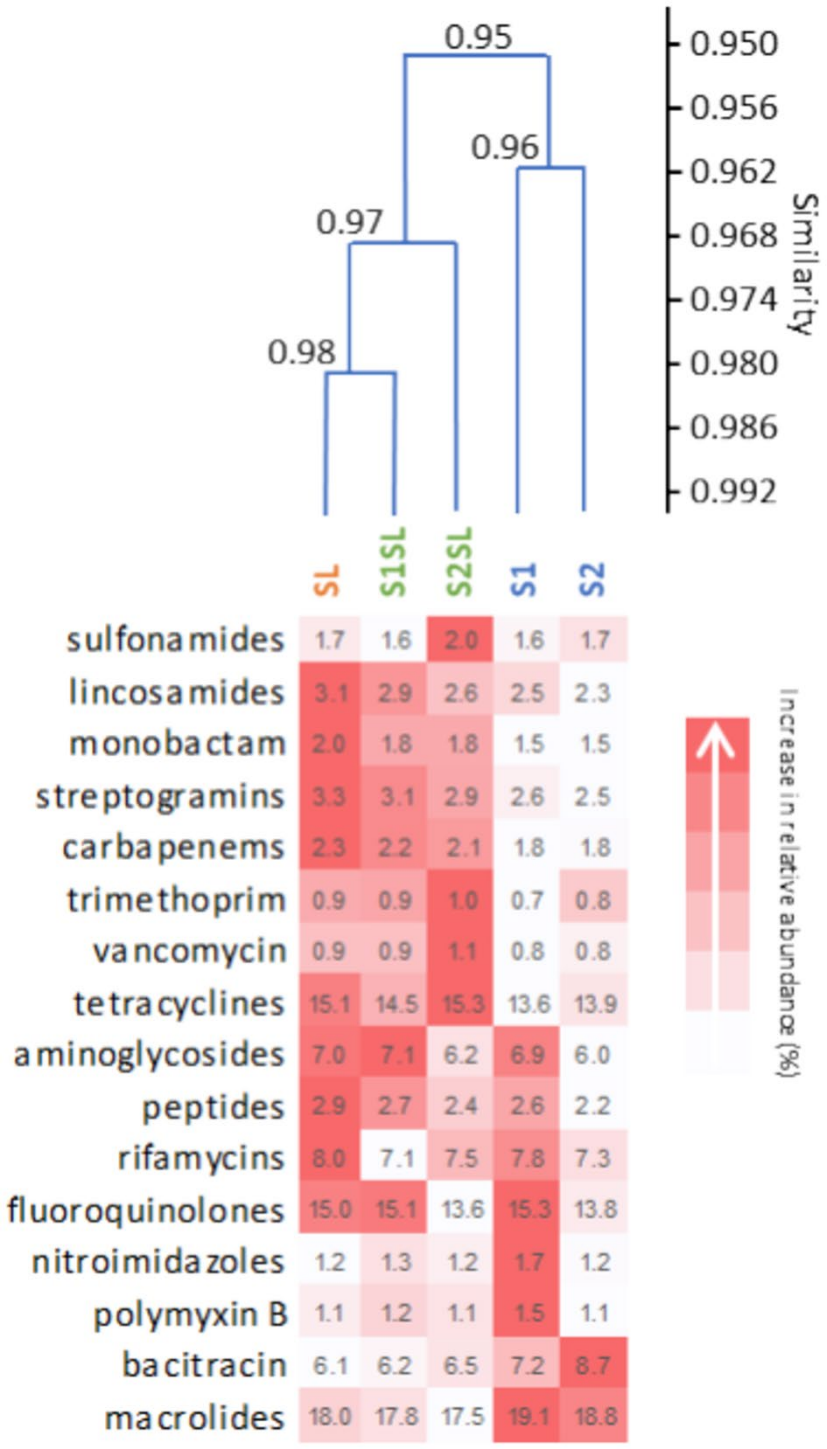
Heat map and cluster analyses describing the differences in relative abundances for antibiotic-resistance genes (ARGs) in the sewage sludge and soil samples. Similarity was estimated according to the Bray–Curtis index (*BC*). The colour scale was set to describe lower to higher values for each group of ARGs. Unfertilized soil samples (S1, S2), fertilized soil samples (S1SL, S2SL) and sewage sludge (SL).

The relative abundances (%) of ARGs were compared between different soil treatments and summarized in Fig. 6. Interestingly, ARGs profiles were characterized by high similitude indices between all samples (*BC* = 0.95–0.98) (Fig. 6). Almost all ARGs displayed the greatest similarity between samples S1SL and SL (0.98), with exception of rifamycin resistance genes (Fig. 6). Sample S2SL also showed high similarity to SL and S1SL (*BC* = 0.97); however, S2SL presented lower abundances of ARGs regarding fluoroquinolones and aminoglycosides compared to S1SL (Fig. 6); in addition, unfertilized S1 and S2 demonstrated lower relative abundances of ARGs than the other three samples. The two control soils differed in the quantity of ARGs conferring resistance to fluoroquinolones, bacitracin and aminoglycosides (Fig. 6).

**Fig. 7:**
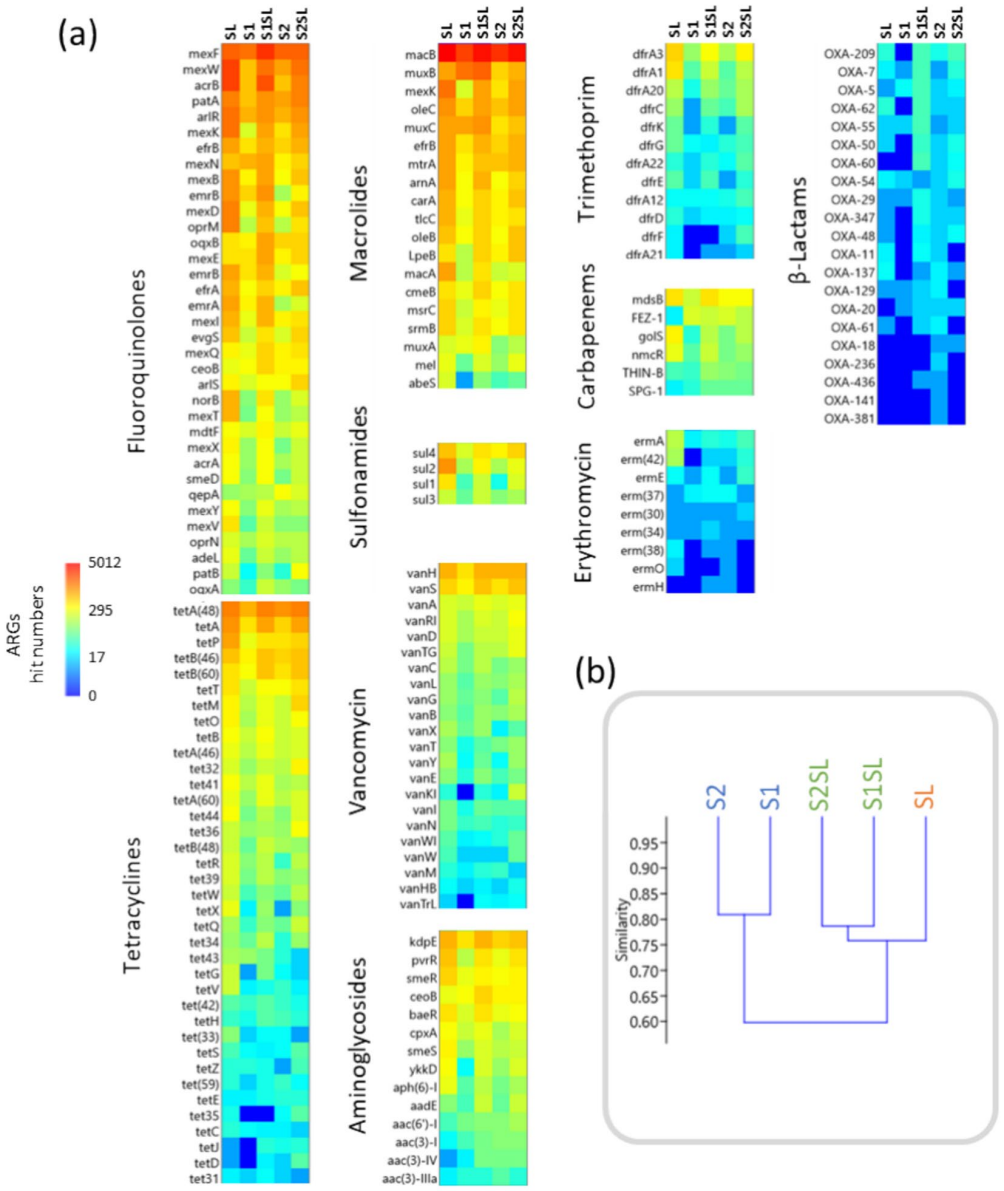
Population of selected ARGs from the constructed metagenomes of experimental soil samples and sewage sludge; (**a**) Heat maps representing the hit numbers of selected ARGs, and (**b**) cluster analysis testing the similarity between samples based on the ARG contents (Bray–Curtis similarity index). Colors represent absolute sequence counts (hit numbers) transformed to Log_10_ values for better visualization.

The hit numbers of seven groups of ARGs were analyzed in Fig. 7a.The presented results include a set of ARGs for which the largest differences were recorded between samples. It is possible to observe a higher number of ARGs in the heat map profiles for SL, S1SL and S2SL when compared to the controls S1 and S2 (Fig. 7a). Fluoroquinolones, tetracyclines and macrolides resistance genes were the most abundant in the constructed metagenomes for all samples. Four genes associated with the resistance to sulfonamides were also present in large numbers. Populations of genes conferring resistance to vancomycin, aminoglycosides and trimethoprim were also found, but in smaller numbers. The least abundant genes in the tested samples were the genes responsible for resistance to β-lactams and erythromycin (Fig. 7a). The vast majority of genes were more numerous or much more numerous in fertilized soil samples S1SL and S2SL compared to unfertilized S1 and S2. Some genes were even more abundant in fertilized soils than in the sludge itself (Fig. 7a). Cluster analysis based on the content of ARGs in the samples was described in Fig. 7b. The results revealed higher similitude between the amended samples S1SL and S2SL with sludge (*BC* = 076), when compared to their respective controls S1 and S2 (*BC* = 0.60) (Fig. 7b).

**Fig. 8:**
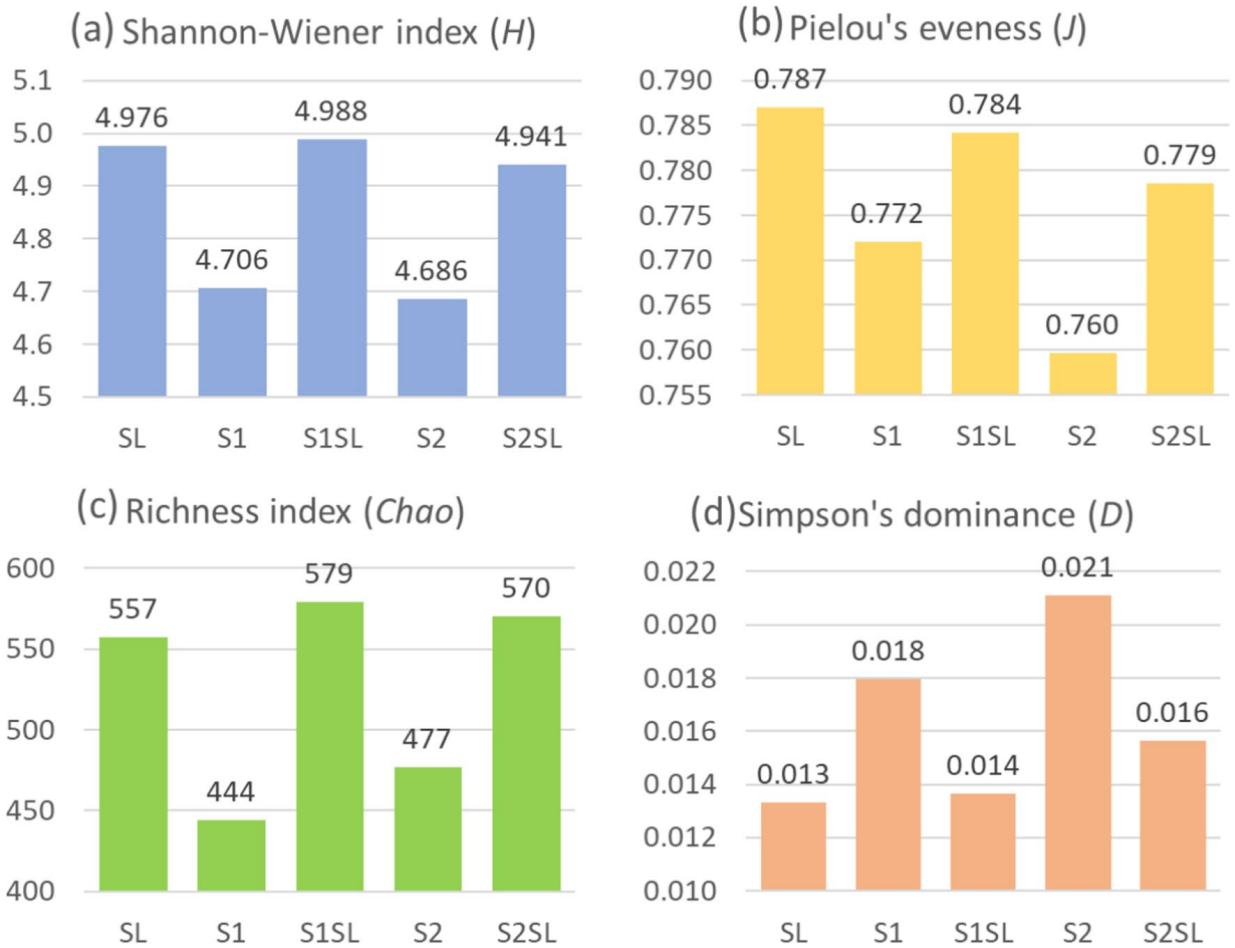
Diversity indices: Shannon–Wiener diversity, Chao-richness, Pielou’s evenness and Simpson’s dominance, according to the relative abundance of antibiotic-resistance genes in the control soil samples (S1, S2), sludge-impacted soil samples (S1SL, S2SL) and sewage sludge (SL).

The diversity, richness and evenness indices according to the relative abundance of antibiotic-resistance genes are presented in Fig. 8a–c. In general, the fertilized soils (S1SL and S2SL) demonstrated higher Shannon Wiener diversity (Fig. 8a), Pielou’s evenness (Fig. 8b) and *Chao* richness (Fig. 8c.) than their respective controls (S1 and S2). Furthermore, all three indices were generally similar between the fertilized soils (S1SL and S2SL) and SL (Fig. 8a–c). These results suggest that the fertilized soils (S1SL and S2SL) demonstrate greater richness and relative ARG abundance than the controls (S1 and S2), which was most likely associated to the fertilization of soils with sludge. The richness index was arguably the most valuable for describing differences between samples, since fertilized soils generally presented up to 135 (S1SL) and 93 (S2SL) exclusive ARGs which were absent from the respective control soils, S1 and S2 (Fig. 8c). In contrast, the Simpson’s dominance index was lower for the fertilized samples (S1SL, S2SL) and sewage sludge (SL) compared to the controls (Fig. 8d), suggesting that the ARGs were more evenly distributed in the fertilized samples S1SL and S2SL, and sewage sludge (SL) than in the controls (S1, S2). This observation was also supported by the fact that a higher evenness index was recorded for the fertilized samples and sludge sediment than the controls (Fig. 8b).

## Discussion

The present study used a metagenomic approach to study the effect of sewage sludge application on soil microbial communities and on the possible spread of ARGs during the seven-weeks field experiment. The two soil types were collected from a rye field (S1) and an onion field (S2), as shown in Table 1; the most significant differences between soils concerned the granulometric properties, pH and the levels of P, Ca and Na ions (Table 1). Our study revealed, that two soils from the different location and of different uses can be characterized by similar proportions of bacterial phyla despite significant differences in soil granulometric properties and pH. The sewage sludge application altered relevantly the structure of soil microbial community compared to unfertilized control soils.

Before fertilization, Planctomycetes and Actinobacteria phyla predominated in both tested soils; these microbes are known to be important components of the soil microbial community, and were the major contributors to the both soils quality before amendment. The Planctomycetes, with dominant species in the present study belonging to *F. ruber*, *G. obscuriglobus*, *P. borealis* and *S. acidiphila* (Fig. 2, Table S3 and Fig. S1), is regarded as the fifth most abundant bacterial phylum in soil that play key roles in global carbon and nitrogen cycles^28,29^.

The Actinobacteria, with dominant species in the present study belonging to *P. medicamentivorans* and *T. album* (Fig. 2, Table S3 and Fig. S1), is another key group of soil bacteria dominant in the rhizosphere, occupying several different ecological niches, including the ability to thrive in harsh environments, nutrient cycling, plant growth promotion, nitrogen fixation, anti-phytopathogenic and saprophytic activity (animal and plant debris decomposition), among others^30,31^. They have the genetic potential for the production of a wide spectrum of secondary metabolites^32–34^; indeed, about 10 000 antibiotics, i.e. 45% of all known bioactive microbial metabolites, have been isolated from Actinobacteria^35^. As such, this phylum has both, considerable ecological and pharmacological potential. A large proportion of Actinobacteria (30%) was found in soil S1 even though the pH was acidic. It has been previously reported that phylum proportions can be strongly influenced by soil pH, especially that Actinobacteria tend to be found in higher proportions in ecosystems, where the pH is relatively high^36^. However, pH is not the only factor that can influence bacterial community composition. In the present study, seven weeks after treatment with sludge, the abundance of these two bacterial phyla decreased seven- to nine-fold in the fertilized soils, which, considering their complex role played in the ecosystem, is highly undesirable (Fig. 1).

Interestingly, the proportions of the Chloroflexi, Proteobacteria and Firmicutes phyla were essentially similar in both S1 and S2 (Fig. 1). The Chloroflexi, a relatively unexplored phylum with the majority of its representatives being unculturable, has shown to be ubiquitous throughout terrestrial to aquatic environments by the use of culture-independent methods^28,37^. They are ecologically and physiologically diverse, and often key agents in oxygen-, nutrient‐, and light‐limited environments^38,39^. Metagenomic analysis has further extended their role in fundamental biogeochemical cycles^40,41^. In our study, the Chloroflexi was the fourth most numerous phylum in both soils (8% in S1 and 11% in S2). However, 50 days after soil fertilization, their relative abundance decreased drastically to a level comparable to that in sewage sludge (0.8% in S1SL, 1.3% in S2SL, 0.5% in SL) (Fig. 1, Table S2).

The Proteobacteria, a group with large proportions for both soils in the present study (13.5% in S1 and 15% in S2; Fig. 1), is a phylum of Gram-negative bacteria prevalent in soil ecosystems with many members associated with a wide range of functions involved in carbon, nitrogen, and sulphur cycling. However, seven weeks after fertilization, their relative abundance drastically increased in both soils (63% in SSL1 and 50% in S2SL), with the appearance of new species, especially among β-P and Δ-Proteobacteria such as *T.denitrificans*, *Thiobacillus* sp. and three species of *Geobacte*r absent previously in unfertilized soils (Fig. 2). Interestingly, they were considerably less abundant in the sludge than in the sludge-impacted S1SL and S2SL (Table S3 and Fig. S1). This indicates that *Geobacter* has adapted well to the soil environment—a genus capable of dissimilatory reduction of Fe(III) oxides, which is an important process for the oxidation of organic matter in terrestrial anoxic environment^42–44^. Furthermore, four species of α-Proteobacteria that were abundant in S1 and S2 (*M. superfactus*, *M. marginalis*, *D. thermohalophilum* and *B. lablabi*) decreased considerably following soil treatment with sludge, reaching similar levels as in sewage sludge (Fig. 2, Table S3 and Fig. S1). This decline in S1SL and S2SL may be due to the presence of harmful components or competitive bacteria in the sewage sludge added to the soil.

Spirochaetes bacteria were not detected in S1 or S2 (Fig. 1), with *Leptonema illini* as the only species observed in fertilized S1SL and S2SL, and sewage sludge. This species found a favorable environment in soils after fertilization, where it multiplied to higher level than in sewage sludge, possibly influenced by sludge components.

Within the phylum Firmicutes, two species dominated in both soils: the spore-forming, thermophilic *Hydrogenibacillus schlegelii* (Fig. 2)—a facultative anaerobe that can oxidize hydrogen aerobically^45^, and the moderately-thermophilic facultative anaerobe *Limnochorda pilosa* (Fig. 2)—capable of denitrification^46^. Approximately 6% of the prokaryotic community of S1 and S2 comprised taxonomic units that could be considered rare taxa, i.e. whose relative abundance was less than 1%. Jiao and Lu^47^ and Xiong et al.^48^, report that the soil and crop system microbiomes comprise a few abundant taxa with a wide niche breadth, and a greater diversity of rare taxa with greater niche specificity. Furthermore, Zhang et al.^49^ report that within each microbial community, rare species played a more important role in driving ecosystem multifunctionality than abundant species.

The presented results could be compared and discussed on the background of other authors interesting research, who have reported that microbial communities in North American soils tend to be dominated by α-Proteobacteria, Acidobacteria and Actinobacteria, as opposed to the Planctomycetes, Bacteroidetes and Firmicutes which, have typically been found to be less abundant^50^.

Sewage sludge had an entirely different taxonomic composition to soils, with a much higher proportion of Proteobacteria (38% in sludge versus 13% in S1 and 15% in S2), Spirochaetes (11%), rare taxa (18%), Halobacteriota (10%) and *Deinococcus* (9%). The PCoA analysis found the addition of sludge composts to soils to result in significant disturbances in the soil microbial community (Fig. 4). Before fertilization, the two soils demonstrated as much as 70% similarity, with this value falling to only 16.6% when comparing S1 with S1SL and 5.8% for S2 and S2SL. The data given in Figs. 1, 2 and 4 suggest that application of sewage sludge had a stronger effect on changes in the bacterial community in S2 than S1.

A soil microbial community comprises a continuum of microorganisms with various carbon requirements. Such bacteria have evolved two distinct ecological strategies according to substrate concentration: oligotrophs favour environments with low levels of nutrients, while copiotrophs (eutrophs) are adapted to live among richer resources. A number of studies suggest that nutrient availability plays an important role in determining the structure and ecological strategy of bacterial communities in nature. Animal-associated microbiota are dominated by copiotrophic bacteria, whereas free-living and plant-associated bacterial communities are mostly dominated by oligotrophs^51–53^. In our study we have adopted the term oligotrophy to estimate bacteria with low requirements in relation to the carbon concentration in their habitat, and copiotrophy to count those with higher requirements. However, this phenomenon is more complex and described in the source literature. The culture method used in the presented study is one of many proposed and described in the literature, though all methods are based on the application of different carbon concentrations in bacteriological media used to differentiate the nutritional requirements of bacterial cells.Although oligotrophs and copiotrophs are ubiquitous in the global ecosystem, their relative abundance and importance in particular ecosystems are still somewhat unclear; therefore, the present study examined the abundance of oligotrophic and copiotrophic bacteria in the analyzed soils. It was found that in both soils, the number of oligotrophic and copiotrophic bacteria increased, and the ratio of oligotrophs to copiotrophs decreased substantially seven weeks after fertilization (Fig. 5). This may indicate that fertilization with sewage sludge may disturb the natural biological balance of the soil, as well as the processes occurring in it, and potential functions of microorganisms as a whole community.

Some studies indicate that around 70% of non-degraded antibiotics are transferred to sewage sludge^54^. Studies on the long-term exposure of environmental bacterial strains to low concentrations of antibiotics indicate that the subinhibitory concentrations of antimicrobials in many environmental samples may influence bacterial ecology, and could drive the selection of antibiotic-resistant bacterial cells. Such selective pressure may also increase the proliferation of ARGs in soil; this is of particular concern, as soil contains numerous bacterial genera of clinical importance^55,56^. Furthermore, antimicrobials and resistant bacteria can shape the diversity and metabolic activity of the soil microbial community, thus negatively affecting the quality of the soil ecosystem^57^. Our findings indicate that the amended soils (S1SL and S2SL) demonstrate greater richness and relative ARG abundance than the controls (S1 and S2), which was most likely related to the fertilization of soils with sewage sludge (Fig. 6; Fig. 8). The most numerous groups of ARGs in the fertilized soils and sewage sludge were those encoding resistance to fluoroquinolones, macrolides, tetracyclines and sulfonamides; this coincides with the list of antibiotics most widely prescribed in human medicine (tetracyclines, macrolides, β-lactams) and veterinary medicine (tetracyclines, sulfonamides, β-lactams)^58–60^. Furthermore, numerous studies have reported the presence of quinolone, tetracycline macrolide, and sulfonamide antibiotics in sewage sludge^61–63^.

Synthetic and semi-synthetic antibiotic residues, such as fluoroquinolones and sulfonamides, possess high chemical stability and resistance to degradation, and are hence often detected in the environment^64,65^. This may explain the high abundance of resistance genes to these antibiotics in the sewage sludge and sludge-fertilized soils used in our experiment. Prolonged exposure to subinhibitory concentrations of antimicrobials may favours the horizontal transfer of drug resistance genes (HGT-horizontal gene transfer) between bacterial cells; this transfer can occur between different species and genera, and between non-pathogenic bacteria and pathogens, resulting in the selection of resistant bacterial cells^10,66–71^. Some vancomycin resistance genes were also found to be more prevalent in the fertilized soil samples than the control soils; higher abundance of these genes is clearly marked in amended soil, even assuming that soil-autochthonous Gram-negative bacteria are, to some extent, naturally resistant to vancomycin due to their cell wall properties. The genes conferring resistance to vancomycin are of particular environmental concern; their concentrations, as well as those of ARGs offering resistance to tetracyclines, macrolides and polymyxin, can increase in sewage sludge after anaerobic digestion in WWTP^72,73^. Furthermore, although β-lactams are among the most frequently prescribed in both, human and veterinary medicine, β-lactam ARGs were not found to be as abundant in our fertilized S1SL and S2SL soils (Fig. 7). The presented results include a set of ARGs for which the largest differences were recorded between samples, excluding a considerable number of those, for which no strong differences were noted. This study opens questions for more detailed analyses with a larger number of sewage sludge from several WWTPs, and the employing of more comprehensive analytical techniques to capture the full spectrum of ARGs and integrons present in the sewage sludge and soil microbial community.

A number of studies has demonstrated that the use of sewage sludge as fertilizer can increase the concentrations of heavy metals in soil^74–77^. As such, Polish regulations specify that sewage sludge used for agricultural purposes must meet certain standards with regard to heavy metal content. However, the level of heavy metals in the sewage sludge used in our study was only a minor percentage of the maximum loads allowed by the Polish regulations^78^, amounting to 6.6% for cadmium, 12.2% for copper, 4.5% for nickel, only 1.8% for lead, 6% for chromium and 25% for zinc. Such low levels did not have a significant effect on their content in the fertilized soils.

The aim of the study was to assess the impact on the soil microbial community and resistome of sewage sludge from a particular municipal WWTP in central Poland, from which sludge is used for fertilizing agricultural soils on a regular basis; the research was conducted as a preliminary, semi-quantitative analysis. The authors are aware that the presented research is limited to an experiment conducted on a single sample of sewage sludge, which may not be representative of other types of sewage sludge used in agriculture. To address these challenges, further study has been undertaken by the authors, that will aim to collect a more diverse range of sewage sludge samples from WWTPs of various sizes, which used different wastewater and sewage sludge treatment methods; the conversion of sewage sludge to a soil fertilizer can be performed by a number of methods, which greatly differ by physicochemical conditions, treatment time, and substrate/amendment composition^79–82^.

The complex structure of sewage sludge, which include antibiotics, microplastics, heavy metals and other composite pollutants, is an important barrier for the removal of ARGs^83^. Although, different treatments have been pointed out to reduce ARB and ARGs in sewage sludge, including stabilization, dewatering, anaerobic digestion, aerobic composting and sludge conditioning^84^, suitable treatment technologies are jet to be identified for specific sludge properties. Anaerobic digestion and aerobic composting were described to be the most effective treatments methods to remove ARGs, however, current methods cannot completely remove the ecological risk. Further solutions improving ARB and ARGs removal include sludge pretreatment, i.e.: addition of coagulants (Fe^3+^, Al^3+^ and free ammonia), thermal hydrolysis, alkaline and ultrasonic methods before anaerobic digestion, or bioleaching and chemical conditioning (Fe^3+^/CaO) before aerobic composting, among others^83^. Several different pre-treatments and modifications of sewage sludge management methods have been recently described, although it was clearly pointed out that proper methods to achieve high ARB and ARGs removal will largely depend on the local sludge composition and properties^83^. Therefore, it is highly recommended to track ARGs in produced sewage sludge to generate predictions for future risk assessment. Furthermore, this could help to identify the most appropriate treatment method for the local composition of sewage sludge prior its use as an agricultural fertilizer.

## Conclusions

This metagenomic study extends the knowledge of soil microbial community and resistome after application of sewage sludge. The research revealed that initially both tested soils, despite differences in their granulometric and physicochemical properties, were dominated by the same bacterial phyla. However, the soils differed in abundance of oligotrophic and copiotrophic bacteria. Our study revealed, that two soils from the different location and of different uses can be characterized by similar proportions of bacterial phyla. Network analysis revealed that the changes occurring in the bacterial structure of both soils were of a similar nature and scale; fertilization tended to favor an increase in Proteobacteria, particularly in β-Proteobacteria and Δ-Proteobacteria in amended soils. Moreover, sewage sludge was a vast repository of ARGs, however it was characterized by low heavy metal content; amendment with sewage sludge negatively affect the richness and relative ARGs abundance in soil. The greatest increase in abundance of ARGs concerned resistance genes to the antibiotics most commonly used in human medicine and veterinary, this may pose a risk to the public health. Our findings provide the data that, alongside other scientific reports, highlight the need to critically revise the use of municipal sewage sludge for agricultural purposes^85–88^. To meet the urgent need for sustainable environmental and agricultural management, modern research techniques that enable a broader approach to soil quality should be widely used. Furthermore, to prevent the ARBs and ARGs contamination of soil, and frame a rational plan of response to this type of environmental threat, it is crucial to identify and track its sources. Studies that are conducted worldwide have regard to different types of soils, distinct climatic zones and different sampling times. Most importantly, the wastewater treatment methods used in different WWTPs vary from country to country and even from one WWTP to another. The method of pretreatment and the dose of sewage sludge applied, are also essential. All of the above factors should be taken into account in comprehensive research, which in turn should be the basis for the elaboration of appropriate, commonly used legal acts.

## Materials and methods

### Soil and sewage sludge characteristics

The study used two types of soil collected from two farmlands located in the Lodz region, Central Poland: S1 from a rye field (latitude—51.917321 N, longitude—19.442669 E) and S2 from an onion field (latitude—52.014598 N, longitude—19.310026 E). The soil sample collection sites were approximately 17 km apart. The sites were farmed to meet the own needs of the farms, and not for commercial purposes. S1 was classified as a sand according to the Soil Taxonomy USDA (United States Department of Agriculture) and PSSS (Polish Society of Soil Science)^89^, and S2 as a loamy sand. The picture of soils was attached as Supplementary Fig. S2.

Three 0–10 cm soil cores were collected from random sites on each farm using sterile polycarbonate tubes, and were composited into one sample to account for soil heterogeneity. The samples were transported on ice to the laboratory, where they were homogenized by hand and stored at 4 °C until analysis. The sewage sludge was collected from the large (class IV) municipal wastewater treatment plant (WWTP) located in central Poland, from which it is used for soil fertilization on a regular basis. The experiment was made in early autumn.

Wastewater treatment plants are graded by size based on population equivalent (p.e.) and wastewater outflow as specified in Polish regulations. A class IV WWTP serves 15,000–99,999 population equivalents—based on the provisions of the Regulation of the Minister of Maritime Economy and Inland Navigation, Journal of Laws, 2019, item 1311, dated 12 July 2019, which implements the provisions of Council Directive 91/271/EEC. The sampled plant is a mechanical and biological sewage treatment plant with chemical support for phosphorus precipitation and the processing of excess sludge generated by autothermal thermophilic sludge stabilization. Its task is to process social, domestic and industrial sewage from the local area, as well as sewage transported by truck from areas not covered by the sewerage system. The particular WWTP is impacted by both industrial (knitting and hosiery) and anthropogenic activity in its catchment.

### Pot experiment

The soil and sludge were crushed and sieved through a 2 mm mesh, the soil was fertilized with sewage sludge at a loading rate of 9 tons per hectare, the dose recommended by Polish Law^90^, then 1 kg of soil, fertilized soil and sewage sludge were loaded into each 1 L pot. The experiment was conducted for seven weeks under field conditions. Samples were collected on day zero for microbiological analysis and again on day 50 for metagenomics and microbiological analysis. Irrigation was performed using distilled and filtered (0.22 µm pore size) water throughout the experiment to avoid introducing extraneous bacterial cells and ARGs into the treated samples (S1SL and S2SL) and samples of soils without sewage sludge (S1 and S2) and sewage sludge alone (SL) as controls.

### Physico-chemical analysis

Organic matter was removed from the samples by combustion of at medium temperature (375–800 °C) in a temperature-regulated muffle furnace^79^, following which, dry ash content was determined. It is important to note that values derived from loss-on-ignition results should only be considered as approximate^91^. The pH was analyzed potentiometrically in H_2_O and 1 mol dm^3^ KCl based on air-dried fine earth samples using a soil/solution ratio of 1:2.5. The pH of the soil was determined potentiometrically in accordance with standards^92^. The available forms of phosphorus (P) and potassium (K**)** were determined using the Egner-Riehm method^93^. The exchangeable forms of magnesium (Mg) were determined using a buffered barium chloride solution (pH 8.1)^82^. Soil carbon (C), hydrogen (H) and nitrogen (N) contents were determined by dry combustion using a CHN elemental analyzer (CHNS, Perkin Elmer, Series II). Exchangeable bases (EB) (i.e., Ca, Mg, K, and Na) were extracted from the soils using 1 mol dm^3^ ammonium chloride at pH 8.2. The samples were washed once using deionized water and water-soluble salts were removed by exchangeable base extractions. Base contents in extracts were measured using flame atomic absorption spectrometry (Ca and Mg) and flame emission spectroscopy (K and Na). Trace element (Cd, Cr, Ni, Zn, Pb, Cu, Cr, Mn, Fe) concentrations were determined after hot digestion in a mixture of HNO_3_ and HClO_4_ (3:2 v/v). The analysis was performed using an inductively-coupled plasma optical emission spectrophotometer (Perkin Elmer ICP-OES Optima 7300 DV) according to US EPA Method 200.7^94^. The quality of the analysis was verified by comparison with a certified reference material (CRM023–050). The recoveries for metals ranged from 89 to 102%. Samples were analyzed in three replicates, for which the relative standard deviations (%RSDs) were less than 10%.

### Microbiological analyses

Briefly, 10 g samples were suspended in 90 ml of autoclaved water and shaken on a rotary shaker at 120 rpm for half an hour at room temperature. A series of dilutions from 10^−1^ to 10^−7^ was prepared in sterile 0.85% NaCl, and 100 µl of each dilution was plated on to media. Microbiological media according to Hattori and Hattori^95^ was used to estimate the number of copiotrophs (NA- nutrient agar) and oligotrophs (NA diluted 100 times in sterile water). The media were supplemented with 50 µg∙ml^−1^ of cycloheximide (Sigma-Aldrich) to avoid fungal growth. The plates were incubated at 25 °C for seven days for copiotrophs and 21 days for oligotrophs before counting. All experiments were performed in triplicate, and all bacterial counts were expressed as colony-forming units (CFU) per 1 g of dry soil or sewage sludge.

### Metagenomic DNA isolation and Shotgun sequencing

Total genomic DNA was extracted from 500 mg (wet mass) of soil and sewage sludge using the FastDNA™ SPIN Kit for Soil (MP Biomedicals, Santa Ana, CA), procedure was conducted according to the manufacturer. The concentration and purity of DNA were estimated by agarose gel electrophoresis and ultraviolet absorbance (Multiscan Sky, Thermo Scientific Inc.). DNA was stored at -20^0^C for further analysis. DNA libraries were processed using the NexteraXT DNA library preparation kit (Illumina) according to the manufacturer’s instructions. The quality of the libraries was assessed on the Agilent Bioanalyzer 2100 system with the Agilent High Sensitivity DNA kit. The libraries were normalized with qPCR using the NEBNext Library Quant Kit for Illumina (New England Biolabs). Metagenomics were performed by shotgun sequencing with the use of the Illumina NovaSeq 6000 platform; the procedure was based on paired-end reads of 2 × 150 bp and a maximum assumption of 40 million paired reads per sample. Original datasets as FASTQ files were uploaded to the NCBI Sequence Read Archive under the project PRJNA1067294, with bio samples for S1 (SAMN39522689—SAMN39522691), S2 (SAMN39522692—SAMN39522694), S1SL (SAMN39522697—SAMN39522699), S2SL (SAMN39522700—SAMN39522702), and SL (SAMN39522695—SAMN39522696).

### Bioinformatic analysis

Sequences were edited before formal analysis. First, they were pre-trimmed to reduce sample contamination, a palindromic algorithm was used for sequence alignment with appropriate adaptors. The trimmed sequences were mined for the presence of antibiotic-resistance genes using the DeepARG tool in the conda environment for Python 2. Short sequences (Shotgun) were analyzed as reads according to Arango-Argoty et al.,^96^. The database hosted by Zenodo (https://zenodo.org/records/8280582) was used to identify ARG-like ORFs with a default condition of probability ≥ 80%. Hit numbers of filtered ARGs were normalized with the 16S rRNA gene using the Kraken2 tool and the results were scaled in a 1:100,000 ratio to optimize their interpretation. Taxonomic units were also classified with Kaiju software to phylum, genus and species level, according to Menzel et al.^97^. Sequences were filtered according to recommended default parameters (length ≥ 11 aa, match score ≥ 65, and e-value ≥ 1 × 10^–2^). The reference database of kaiju proGenomes v3 was chosen for taxonomical characterization (https://progenomes.embl.de). For the purpose of the present study, only sequences belonging to bacteria and archaea were considered for analysis.

### Analysis of data

Bacterial and archaeal taxonomic data was visualized with the phyloseq package. The taxa that were exclusive to each sample, and those that were shared, were plotted in venn diagrams with the ggvenn package, both in R environment. Furthermore, distance between sample replicates was visualized with principal coordinate analysis (PCoA) according to the taxonomical composition. Significant differences between samples were tested with the Kruskal–Wallis and Mann–Whitney test pairwise. Alpha diversity indices, including Shannon–Wiener (*H*), Richness (*Chao*), Pielou’s eveness (*J*) and Simpson’s dominance (*D*), were used to describe the composition of ARGs in the samples. Finally, cluster analysis based on the Bray Curtis similarity index was used to visualize the differences in ARG composition and metal concentration between samples. The PCoA, statistical differences, diversity and cluster analyses were performed with PAST 4.12 software^98^.

Co-occurrence network analysis was performed to investigate the relationship between microbial communities at different levels of treatment. The network was constructed and visualized with the software Cytoscape 3.10.2 (https://cytoscape.org/), focusing on prokaryote interactions with high and significant Spearman’s correlations (*r*_*s*_ > 0.56 and *p* < 0.05). The significance (*p* values) was adjusted for multiple testing with Bonferroni correction. Topological features were used to construct the network, including nodes as an estimable species marker (prokaryote) and edges as the correlation weight between two defined nodes. Positive and negative correlations between nodes were used to identify key taxa that were replaced or enriched in amended soils (S1SL and S2SL), when compared to sewage sludge (SL) and the control soils (S1 and S2).

## Supplementary Information

Supplementary Information.

## Data Availability

Original datasets as FASTQ files were uploaded to the NCBI Sequence Read Archive under the project PRJNA1067294, with bio samples for S1 (SAMN39522689—SAMN39522691), S2 (SAMN39522692—SAMN39522694), S1SL (SAMN39522697—SAMN39522699), S2SL (SAMN39522700—SAMN39522702), and SL (SAMN39522695—SAMN39522696).
