# Supplementary material for: Sewage sludge fertilization affects microbial community structure and its resistome in agricultural soils
Source: Sci Rep. 2024 Sep 9;14:21034. doi: 10.1038/s41598-024-71656-0 (PMC11385149; doi:10.1038/s41598-024-71656-0)
Supplement: Supplementary file 1 — Supplementary Information. [file 41598_2024_71656_MOESM1_ESM.docx]

**Sewage sludge fertilization affects microbial community structure and its resistome in agricultural soils**

Serwecińska L.*, Font- Nájera A., Strapagiel D., Lach J., Tołoczko W., Bołdak M., Urbaniak M.

1 European Regional Centre for Ecohydrology of the Polish Academy of Sciences, Tylna 3, 90‑364 Lodz, Poland

2 Biobank Lab, Department of Oncobiology and Epigenetics, Faculty of Biology and Environmental Protection, University of Lodz, Pomorska 139, 91-402 Lodz, Poland

3 Department of Physical Geography, Faculty of Geographical Sciences, University of Lodz, Narutowicza 88, 90-139 Lodz, Poland

4 Department of Agriculture and Environmental Chemistry, University of Agriculture in Krakow, Mickiewicza 21, 31-120 Krakow, Poland

5 UNESCO Chair On Ecohydrology and Applied Ecology, Faculty of Biology and Environmental Protection, University of Lodz, Banacha 12/16, 90‑237 Lodz, Poland

*Corresponding author: L. Serwecińska ([l.serwecinska@erce](mailto:l.serwecinska@erce).unesco.lodz.pl)

**Supplementary material**

**Table S1.** Properties of sewage sludge used in the present study.

| **pH** | **Dry mass** | **TOC** | **Total N** | **N-NH_4_** | **Total P** | **K^+^** | **Ca^2+^** | **Mg^2+^** |
| --- | --- | --- | --- | --- | --- | --- | --- | --- |
| (H_2_O) | % | % d.m. | % d.m. | % d.m. | % d.m. | % d.m. | % d.m. | % d.m. |
| 8.13 | 28.35 | 56.83 | 7.55 | 0.76 | 2.62 | n.d. | 3.73 | 1.03 |

N: nitrogen, P: phosphorus, d.m.: dry mass; n.d.: not detected.

**Table S2**. Description of relative abundances (%) of prokaryotic taxa in the different sediment samples at the level of phylum.

| **Phylum** | **SL** | **S1** | **S1SL** | **S2** | **S2SL** |
| --- | --- | --- | --- | --- | --- |
| Halobacteriota | 10.5 | 0.0 | 0.7 | 0.0 | 0.4 |
| Actinobacteria | 3.6 | 29.2 | 3.7 | 14.2 | 2.1 |
| Bacteroidetes | 2.5 | 0.0 | 1.2 | 0.0 | 3.7 |
| Chloroflexi | 0.5 | 8.2 | 0.8 | 11.0 | 1.3 |
| Deinococcus | 8.4 | 0.0 | 0.3 | 0.0 | 0.8 |
| Firmicutes | 1.4 | 5.0 | 0.9 | 6.3 | 1.2 |
| Planctomycetes | 3.2 | 38.3 | 4.4 | 47.8 | 7.8 |
| Proteobacteria | 36.9 | 13.3 | 62.5 | 14.7 | 49.8 |
| Spirochaetes | 11.1 | 0.0 | 17.5 | 0.0 | 18.9 |
| Tenericutes | 3.8 | 0.0 | 0.3 | 0.0 | 3.1 |
| Others | 18.2 | 5.8 | 7.8 | 5.9 | 11.0 |

**Table S3**. Description of relative abundances (%) of prokaryotic taxa in the different sediment samples at the level of species.

| **Phylum** | **Species** | **c** | **S1** | **S1SL** | **S2** | **S2SL** |
| --- | --- | --- | --- | --- | --- | --- |
| Halobacteriota | *Methanosarcina_mazei* | 10.5 | 0.0 | 0.7 | 0.0 | 0.4 |
| Actinobacteria | *Mycolicibacterium_fallax* | 2.9 | 0.0 | 0.9 | 0.0 | 0.4 |
| Actinobacteria | *Patulibacter_medicamentivorans* | 0.2 | 7.4 | 0.7 | 1.9 | 0.3 |
| Actinobacteria | *Thermoleophilum_album* | 0.5 | 21.8 | 2.1 | 12.4 | 1.4 |
| Bacteroidetes | *Bacteroidales_bacterium_6E* | 0.3 | 0.0 | 0.0 | 0.0 | 1.0 |
| Bacteroidetes | *Fermentimonas_caenicola* | 0.7 | 0.0 | 0.7 | 0.0 | 1.1 |
| Bacteroidetes | *Marinilabiliaceae_bacterium* | 1.6 | 0.0 | 0.5 | 0.0 | 1.6 |
| Chloroflexi | *Kouleothrix_aurantiaca* | 0.4 | 3.2 | 0.4 | 3.6 | 0.7 |
| Chloroflexi | *Nitrolancea_hollandica* | 0.1 | 5.0 | 0.4 | 7.4 | 0.6 |
| Deinococcus | *Thermus_thermophilus* | 8.4 | 0.0 | 0.3 | 0.0 | 0.8 |
| Firmicutes | *Hydrogenibacillus_schlegelii* | 0.6 | 0.7 | 0.2 | 2.6 | 0.5 |
| Firmicutes | *Limnochorda_pilosa* | 0.8 | 4.3 | 0.6 | 3.7 | 0.7 |
| Planctomycetes | *Fimbriiglobus_ruber* | 0.4 | 20.8 | 2.2 | 27.6 | 3.7 |
| Planctomycetes | *Gemmata_obscuriglobus* | 0.9 | 5.1 | 1.1 | 7.3 | 1.7 |
| Planctomycetes | *Paludisphaera_borealis* | 1.5 | 8.6 | 0.8 | 9.7 | 1.8 |
| Planctomycetes | *Singulisphaera_acidiphila* | 0.3 | 3.8 | 0.3 | 3.2 | 0.5 |
| Proteobacteria | *Bradyrhizobium_lablabi* | 0.0 | 1.6 | 0.1 | 0.0 | 0.2 |
| Proteobacteria | *Dichotomicrobium_thermohalophilum* | 0.6 | 2.0 | 0.4 | 0.9 | 0.4 |
| Proteobacteria | *Methyloceanibacter_marginalis* | 0.3 | 2.3 | 0.3 | 2.0 | 0.3 |
| Proteobacteria | *Methyloceanibacter_superfactus* | 0.4 | 3.6 | 0.5 | 3.1 | 0.4 |
| Proteobacteria | *Parvibaculum_sp* | 1.2 | 0.0 | 0.2 | 0.0 | 0.0 |
| Proteobacteria | *Ferrovibrio_sp* | 0.0 | 0.0 | 2.2 | 0.0 | 0.3 |
| Proteobacteria | *Sphingopyxis_granuli* | 5.2 | 0.0 | 0.2 | 0.0 | 0.0 |
| Proteobacteria | *Lautropia_sp_SCN_69_89* | 0.2 | 0.0 | 1.9 | 0.0 | 1.1 |
| Proteobacteria | *Alicycliphilus_denitrificans* | 2.2 | 0.0 | 1.6 | 0.0 | 1.1 |
| Proteobacteria | *Brachymonas_denitrificans* | 7.0 | 0.0 | 0.4 | 0.0 | 0.4 |
| Proteobacteria | *Comamonas_sp_SCN_65_56* | 1.8 | 0.0 | 0.0 | 0.0 | 0.0 |
| Proteobacteria | *Sutterella_parvirubra* | 1.1 | 2.1 | 0.7 | 3.5 | 1.0 |
| Proteobacteria | *Methylobacillus_sp_MM3* | 0.0 | 0.0 | 0.0 | 0.6 | 1.3 |
| Proteobacteria | *Nitrosomonas_eutropha* | 1.1 | 0.0 | 0.0 | 0.0 | 0.0 |
| Proteobacteria | *Sterolibacterium_denitrificans* | 1.6 | 0.0 | 0.4 | 0.0 | 0.2 |
| Proteobacteria | *Thiobacillus_denitrificans* | 9.1 | 0.0 | 4.7 | 0.0 | 4.8 |
| Proteobacteria | *Thiobacillus_sp_65_29* | 1.1 | 0.0 | 3.7 | 0.0 | 3.3 |
| Proteobacteria | *Rhodocyclales_bacterium* | 0.0 | 0.0 | 0.2 | 0.0 | 1.6 |
| Proteobacteria | *Geobacter_anodireducens* | 0.4 | 0.0 | 25.4 | 0.0 | 16.7 |
| Proteobacteria | *Geobacter_soli* | 0.2 | 0.0 | 11.7 | 0.0 | 8.7 |
| Proteobacteria | *Geobacter_sulfurreducens* | 0.0 | 0.0 | 5.5 | 0.0 | 3.5 |
| Proteobacteria | *Nannocystis_exedens* | 0.1 | 1.7 | 1.3 | 4.5 | 3.0 |
| Proteobacteria | *Acinetobacter_towneri* | 1.2 | 0.0 | 0.0 | 0.0 | 0.5 |
| Proteobacteria | *Stenotrophomonas_koreensis* | 1.4 | 0.0 | 0.0 | 0.0 | 0.0 |
| Proteobacteria | *Stenotrophomonas_rhizophila* | 0.6 | 0.0 | 1.3 | 0.2 | 1.1 |
| Spirochaetes | *Leptonema_illini* | 11.1 | 0.0 | 17.5 | 0.0 | 18.9 |
| Tenericutes | *Acholeplasma_laidlawii* | 2.3 | 0.0 | 0.0 | 0.0 | 0.9 |
| Tenericutes | *Acholeplasma_oculi* | 1.5 | 0.0 | 0.2 | 0.0 | 2.2 |
| Others | | 18.2 | 5.8 | 7.8 | 5.9 | 11.0 |


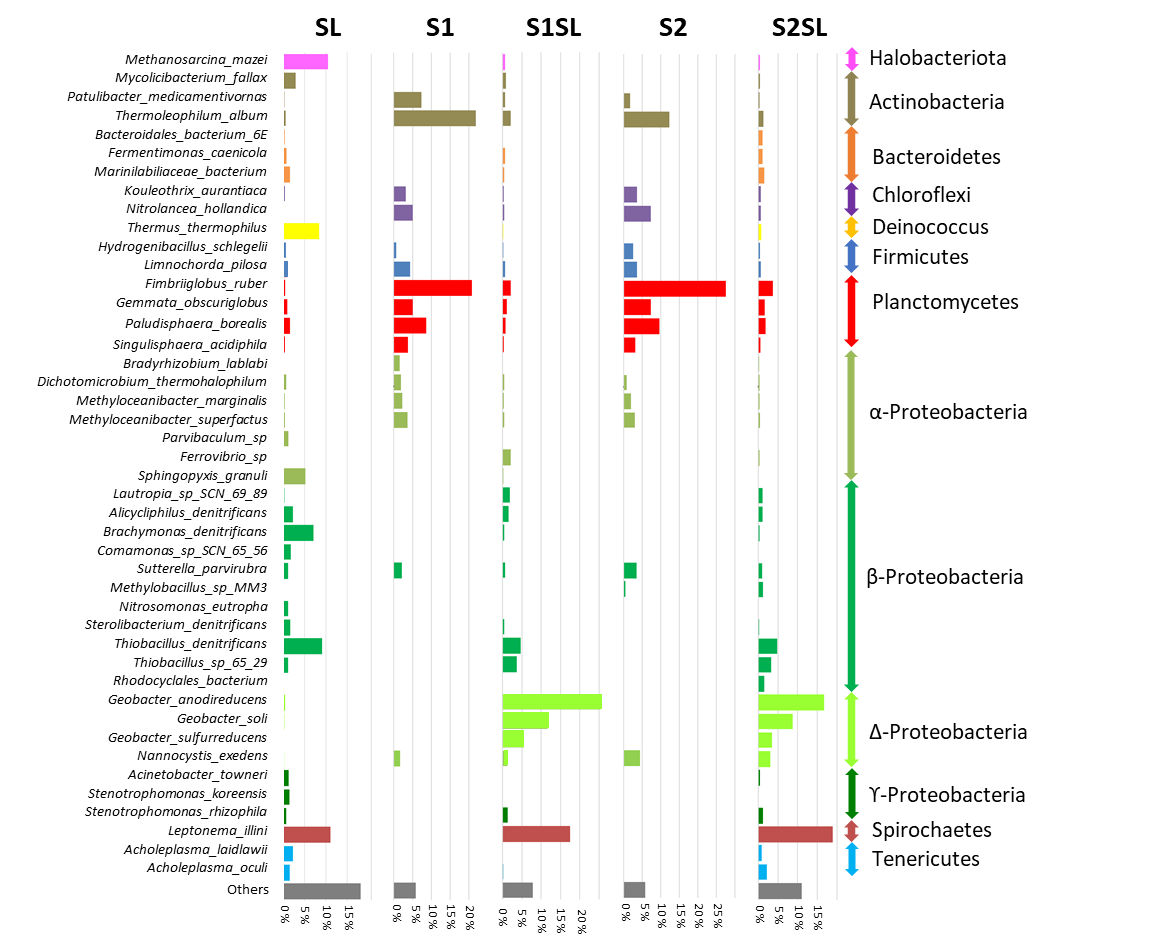


**Fig S1.** The species-level composition of prokaryotic microbiome for sewage sludge (SL), unamended soil samples (S1, S2) and soils amended with sewage sludge (S1SL, S2SL). The graph describes only the taxonomic units that were >1% of total abundance. All remaining taxa, showing <1% of total abundance, are given in the category *others*.

**Table S4.** Description of topological features for the construction of network analysis of prokaryotic assemblages between treatments using Spearman’s correlation (*r_s_*) and *p* significant values after Bonferroni correction (1/6).

| **POSITIVE CORRELATIONS** | | | | | | | | |
| --- | --- | --- | --- | --- | --- | --- | --- | --- |
| **Base node** | | | **Target node** | | | **Edge parameters** | | |
| **no.** | **Taxa** | **Cluster** | **no.** | **Taxa** | **Cluster** | ***r_s_*** | **S** | ***p*** |
| 1 | Comamonas_sp_SCN_65_56 | SL | 5 | Sphingopyxis_granuli | SL | 1.00 | 4 | 9.3E-16 |
| 1 | Comamonas_sp_SCN_65_56 | SL | 7 | Methanosarcina_mazei | SL | 0.99 | 4 | 4.3E-11 |
| 1 | Comamonas_sp_SCN_65_56 | SL | 4 | Brachymonas_denitrificans | SL | 0.99 | 4 | 3.3E-10 |
| 1 | Comamonas_sp_SCN_65_56 | SL | 6 | Parvibaculum_sp | SL | 0.98 | 4 | 2.3E-08 |
| 1 | Comamonas_sp_SCN_65_56 | SL | 9 | Thermus_thermophilus | SL | 0.96 | 4 | 4.1E-07 |
| 1 | Comamonas_sp_SCN_65_56 | SL | 37 | Mycolicibacterium_fallax | S1SL S2SL | 0.86 | 2 | 3.1E-04 |
| 2 | Stenotrophomonas_koreensis | SL | 3 | Nitrosomonas_eutropha | SL | 1.00 | 4 | 3.9E-19 |
| 2 | Stenotrophomonas_koreensis | SL | 1 | Comamonas_sp_SCN_65_56 | SL | 1.00 | 4 | 3.3E-14 |
| 2 | Stenotrophomonas_koreensis | SL | 5 | Sphingopyxis_granuli | SL | 1.00 | 4 | 3.3E-13 |
| 2 | Stenotrophomonas_koreensis | SL | 7 | Methanosarcina_mazei | SL | 1.00 | 4 | 2.2E-11 |
| 2 | Stenotrophomonas_koreensis | SL | 4 | Brachymonas_denitrificans | SL | 0.99 | 4 | 2.2E-10 |
| 2 | Stenotrophomonas_koreensis | SL | 6 | Parvibaculum_sp | SL | 0.98 | 4 | 5.6E-08 |
| 2 | Stenotrophomonas_koreensis | SL | 9 | Thermus_thermophilus | SL | 0.97 | 4 | 1.4E-07 |
| 2 | Stenotrophomonas_koreensis | SL | 8 | Sterolibacterium_denitrificans | SL | 0.90 | 2 | 8.2E-05 |
| 2 | Stenotrophomonas_koreensis | SL | 37 | Mycolicibacterium_fallax | S1SL S2SL | 0.85 | 2 | 5.3E-04 |
| 2 | Stenotrophomonas_koreensis | SL | 43 | Acinetobacter_towneri | S2SL | 0.66 | 1 | 2.0E-02 |
| 3 | Nitrosomonas_eutropha | SL | 1 | Comamonas_sp_SCN_65_56 | SL | 1.00 | 4 | 1.7E-14 |
| 3 | Nitrosomonas_eutropha | SL | 5 | Sphingopyxis_granuli | SL | 1.00 | 4 | 2.8E-13 |
| 3 | Nitrosomonas_eutropha | SL | 7 | Methanosarcina_mazei | SL | 0.99 | 4 | 4.9E-11 |
| 3 | Nitrosomonas_eutropha | SL | 4 | Brachymonas_denitrificans | SL | 0.99 | 4 | 5.9E-10 |
| 3 | Nitrosomonas_eutropha | SL | 6 | Parvibaculum_sp | SL | 0.98 | 4 | 5.8E-08 |
| 3 | Nitrosomonas_eutropha | SL | 9 | Thermus_thermophilus | SL | 0.97 | 4 | 2.6E-07 |
| 3 | Nitrosomonas_eutropha | SL | 37 | Mycolicibacterium_fallax | S1SL S2SL | 0.85 | 2 | 5.1E-04 |
| 4 | Brachymonas_denitrificans | SL | 7 | Methanosarcina_mazei | SL | 1.00 | 4 | 2.7E-12 |
| 4 | Brachymonas_denitrificans | SL | 5 | Sphingopyxis_granuli | SL | 0.99 | 4 | 2.0E-10 |
| 4 | Brachymonas_denitrificans | SL | 9 | Thermus_thermophilus | SL | 0.99 | 4 | 3.3E-09 |
| 4 | Brachymonas_denitrificans | SL | 6 | Parvibaculum_sp | SL | 0.98 | 4 | 3.1E-08 |
| 4 | Brachymonas_denitrificans | SL | 37 | Mycolicibacterium_fallax | S1SL S2SL | 0.87 | 2 | 2.2E-04 |
| 4 | Brachymonas_denitrificans | SL | 36 | Alicycliphilus_denitrificans | S1SL S2SL | 0.59 | 1 | 4.4E-02 |
| 5 | Sphingopyxis_granuli | SL | 7 | Methanosarcina_mazei | SL | 1.00 | 4 | 6.7E-12 |
| 5 | Sphingopyxis_granuli | SL | 6 | Parvibaculum_sp | SL | 0.99 | 4 | 3.5E-09 |
| 5 | Sphingopyxis_granuli | SL | 9 | Thermus_thermophilus | SL | 0.96 | 4 | 4.2E-07 |
| 5 | Sphingopyxis_granuli | SL | 37 | Mycolicibacterium_fallax | S1SL S2SL | 0.87 | 2 | 2.4E-04 |
| 6 | Parvibaculum_sp | SL | 7 | Methanosarcina_mazei | SL | 0.99 | 4 | 2.2E-09 |
| 6 | Parvibaculum_sp | SL | 9 | Thermus_thermophilus | SL | 0.95 | 3 | 3.5E-06 |
| 6 | Parvibaculum_sp | SL | 37 | Mycolicibacterium_fallax | S1SL S2SL | 0.90 | 3 | 6.2E-05 |
| 8 | Sterolibacterium_denitrificans | SL | 6 | Parvibaculum_sp | SL | 0.95 | 3 | 3.4E-06 |
| 8 | Sterolibacterium_denitrificans | SL | 4 | Brachymonas_denitrificans | SL | 0.94 | 3 | 6.7E-06 |
| 8 | Sterolibacterium_denitrificans | SL | 7 | Methanosarcina_mazei | SL | 0.93 | 3 | 1.2E-05 |
| 8 | Sterolibacterium_denitrificans | SL | 9 | Thermus_thermophilus | SL | 0.93 | 3 | 1.3E-05 |
| 8 | Sterolibacterium_denitrificans | SL | 5 | Sphingopyxis_granuli | SL | 0.91 | 3 | 3.7E-05 |
| 8 | Sterolibacterium_denitrificans | SL | 1 | Comamonas_sp_SCN_65_56 | SL | 0.90 | 3 | 6.2E-05 |
| 8 | Sterolibacterium_denitrificans | SL | 37 | Mycolicibacterium_fallax | S1SL S2SL | 0.89 | 2 | 8.8E-05 |
| 8 | Sterolibacterium_denitrificans | SL | 3 | Nitrosomonas_eutropha | SL | 0.89 | 2 | 1.0E-04 |
| 8 | Sterolibacterium_denitrificans | SL | 36 | Alicycliphilus_denitrificans | S1SL S2SL | 0.80 | 2 | 1.6E-03 |
| 9 | Thermus_thermophilus | SL | 7 | Methanosarcina_mazei | SL | 0.98 | 4 | 3.5E-08 |
| 9 | Thermus_thermophilus | SL | 37 | Mycolicibacterium_fallax | S1SL S2SL | 0.81 | 2 | 1.3E-03 |
| 10 | Fimbriiglobus_ruber | S1 S2 | 14 | Kouleothrix_aurantiaca | S1 S2 | 0.99 | 4 | 1.5E-10 |
| 10 | Fimbriiglobus_ruber | S1 S2 | 11 | Nitrolancea_hollandica | S1 S2 | 0.97 | 4 | 1.0E-07 |
| 10 | Fimbriiglobus_ruber | S1 S2 | 17 | Limnochorda_pilosa | S1 S2 | 0.91 | 3 | 3.7E-05 |
| 10 | Fimbriiglobus_ruber | S1 S2 | 18 | Thermoleophilum_album | S1 | 0.82 | 2 | 1.0E-03 |
| 10 | Fimbriiglobus_ruber | S1 S2 | 23 | Hydrogenibacillus_schlegelii | S1 S2 | 0.77 | 1 | 3.2E-03 |
| 11 | Nitrolancea_hollandica | S1 S2 | 14 | Kouleothrix_aurantiaca | S1 S2 | 0.97 | 4 | 1.4E-07 |
| 11 | Nitrolancea_hollandica | S1 S2 | 18 | Thermoleophilum_album | S1 | 0.75 | 1 | 4.6E-03 |
| 12 | Paludisphaera_borealis | S1 S2 | 13 | Gemmata_obscuriglobus | S1 S2 | 0.95 | 3 | 1.9E-06 |
| 12 | Paludisphaera_borealis | S1 S2 | 10 | Fimbriiglobus_ruber | S1 S2 | 0.93 | 3 | 1.0E-05 |
| 12 | Paludisphaera_borealis | S1 S2 | 14 | Kouleothrix_aurantiaca | S1 S2 | 0.92 | 3 | 2.3E-05 |
| 12 | Paludisphaera_borealis | S1 S2 | 17 | Limnochorda_pilosa | S1 S2 | 0.88 | 2 | 1.6E-04 |
| 12 | Paludisphaera_borealis | S1 S2 | 18 | Thermoleophilum_album | S1 | 0.85 | 2 | 4.4E-04 |
| 12 | Paludisphaera_borealis | S1 S2 | 11 | Nitrolancea_hollandica | S1 S2 | 0.84 | 2 | 6.4E-04 |
| 12 | Paludisphaera_borealis | S1 S2 | 23 | Hydrogenibacillus_schlegelii | S1 S2 | 0.71 | 1 | 9.6E-03 |
| 12 | Paludisphaera_borealis | S1 S2 | 21 | Patulibacter_medicamentivorans | S1 | 0.62 | 1 | 3.0E-02 |
| 13 | Gemmata_obscuriglobus | S1 S2 | 10 | Fimbriiglobus_ruber | S1 S2 | 0.92 | 3 | 1.8E-05 |
| 13 | Gemmata_obscuriglobus | S1 S2 | 14 | Kouleothrix_aurantiaca | S1 S2 | 0.89 | 2 | 9.7E-05 |
| 13 | Gemmata_obscuriglobus | S1 S2 | 23 | Hydrogenibacillus_schlegelii | S1 S2 | 0.86 | 2 | 3.8E-04 |
| 13 | Gemmata_obscuriglobus | S1 S2 | 11 | Nitrolancea_hollandica | S1 S2 | 0.83 | 2 | 8.1E-04 |
| 13 | Gemmata_obscuriglobus | S1 S2 | 17 | Limnochorda_pilosa | S1 S2 | 0.82 | 2 | 1.0E-03 |
| 13 | Gemmata_obscuriglobus | S1 S2 | 18 | Thermoleophilum_album | S1 | 0.76 | 1 | 4.4E-03 |

**Table S4.** Continuation (2/6).

| **POSITIVE CORRELATIONS** | | | | | | | | |
| --- | --- | --- | --- | --- | --- | --- | --- | --- |
| **Base node** | | | **Target node** | | | **Edge parameters** | | |
| **no.** | **Taxa** | **Cluster** | **no.** | **Taxa** | **Cluster** | ***r_s_*** | **S** | ***p*** |
| 14 | Kouleothrix_aurantiaca | S1 S2 | 18 | Thermoleophilum_album | S1 | 0.86 | 2 | 3.0E-04 |
| 14 | Kouleothrix_aurantiaca | S1 S2 | 21 | Patulibacter_medicamentivorans | S1 | 0.63 | 1 | 2.8E-02 |
| 15 | Methyloceanibacter_marginalis | S1 S2 | 16 | Singulisphaera_acidiphila | S1 S2 | 0.97 | 4 | 2.8E-07 |
| 15 | Methyloceanibacter_marginalis | S1 S2 | 12 | Paludisphaera_borealis | S1 S2 | 0.95 | 3 | 3.2E-06 |
| 15 | Methyloceanibacter_marginalis | S1 S2 | 18 | Thermoleophilum_album | S1 | 0.94 | 3 | 6.4E-06 |
| 15 | Methyloceanibacter_marginalis | S1 S2 | 14 | Kouleothrix_aurantiaca | S1 S2 | 0.92 | 3 | 2.0E-05 |
| 15 | Methyloceanibacter_marginalis | S1 S2 | 10 | Fimbriiglobus_ruber | S1 S2 | 0.91 | 3 | 3.4E-05 |
| 15 | Methyloceanibacter_marginalis | S1 S2 | 13 | Gemmata_obscuriglobus | S1 S2 | 0.90 | 3 | 6.1E-05 |
| 15 | Methyloceanibacter_marginalis | S1 S2 | 17 | Limnochorda_pilosa | S1 S2 | 0.87 | 2 | 2.1E-04 |
| 15 | Methyloceanibacter_marginalis | S1 S2 | 11 | Nitrolancea_hollandica | S1 S2 | 0.84 | 2 | 6.6E-04 |
| 15 | Methyloceanibacter_marginalis | S1 S2 | 20 | Dichotomicrobium_thermohalophilum | S1 | 0.83 | 2 | 8.0E-04 |
| 15 | Methyloceanibacter_marginalis | S1 S2 | 21 | Patulibacter_medicamentivorans | S1 | 0.76 | 1 | 4.4E-03 |
| 15 | Methyloceanibacter_marginalis | S1 S2 | 23 | Hydrogenibacillus_schlegelii | S1 S2 | 0.68 | 1 | 1.6E-02 |
| 16 | Singulisphaera_acidiphila | S1 S2 | 18 | Thermoleophilum_album | S1 | 0.96 | 3 | 1.1E-06 |
| 16 | Singulisphaera_acidiphila | S1 S2 | 17 | Limnochorda_pilosa | S1 S2 | 0.95 | 3 | 1.4E-06 |
| 16 | Singulisphaera_acidiphila | S1 S2 | 14 | Kouleothrix_aurantiaca | S1 S2 | 0.95 | 3 | 2.2E-06 |
| 16 | Singulisphaera_acidiphila | S1 S2 | 12 | Paludisphaera_borealis | S1 S2 | 0.94 | 3 | 4.2E-06 |
| 16 | Singulisphaera_acidiphila | S1 S2 | 10 | Fimbriiglobus_ruber | S1 S2 | 0.93 | 3 | 1.0E-05 |
| 16 | Singulisphaera_acidiphila | S1 S2 | 13 | Gemmata_obscuriglobus | S1 S2 | 0.89 | 2 | 9.2E-05 |
| 16 | Singulisphaera_acidiphila | S1 S2 | 11 | Nitrolancea_hollandica | S1 S2 | 0.86 | 2 | 3.5E-04 |
| 16 | Singulisphaera_acidiphila | S1 S2 | 21 | Patulibacter_medicamentivorans | S1 | 0.79 | 1 | 2.2E-03 |
| 16 | Singulisphaera_acidiphila | S1 S2 | 23 | Hydrogenibacillus_schlegelii | S1 S2 | 0.62 | 1 | 3.1E-02 |
| 17 | Limnochorda_pilosa | S1 S2 | 14 | Kouleothrix_aurantiaca | S1 S2 | 0.94 | 3 | 6.6E-06 |
| 17 | Limnochorda_pilosa | S1 S2 | 18 | Thermoleophilum_album | S1 | 0.91 | 3 | 3.5E-05 |
| 17 | Limnochorda_pilosa | S1 S2 | 11 | Nitrolancea_hollandica | S1 S2 | 0.87 | 2 | 2.3E-04 |
| 17 | Limnochorda_pilosa | S1 S2 | 21 | Patulibacter_medicamentivorans | S1 | 0.76 | 1 | 4.2E-03 |
| 18 | Thermoleophilum_album | S1 | 21 | Patulibacter_medicamentivorans | S1 | 0.93 | 3 | 1.5E-05 |
| 19 | Methyloceanibacter_superfactus | S1 | 15 | Methyloceanibacter_marginalis | S1 S2 | 0.95 | 3 | 2.8E-06 |
| 19 | Methyloceanibacter_superfactus | S1 | 16 | Singulisphaera_acidiphila | S1 S2 | 0.94 | 3 | 5.8E-06 |
| 19 | Methyloceanibacter_superfactus | S1 | 13 | Gemmata_obscuriglobus | S1 S2 | 0.90 | 3 | 5.9E-05 |
| 19 | Methyloceanibacter_superfactus | S1 | 18 | Thermoleophilum_album | S1 | 0.90 | 2 | 7.2E-05 |
| 19 | Methyloceanibacter_superfactus | S1 | 12 | Paludisphaera_borealis | S1 S2 | 0.88 | 2 | 1.4E-04 |
| 19 | Methyloceanibacter_superfactus | S1 | 14 | Kouleothrix_aurantiaca | S1 S2 | 0.85 | 2 | 5.1E-04 |
| 19 | Methyloceanibacter_superfactus | S1 | 17 | Limnochorda_pilosa | S1 S2 | 0.84 | 2 | 6.2E-04 |
| 19 | Methyloceanibacter_superfactus | S1 | 10 | Fimbriiglobus_ruber | S1 S2 | 0.84 | 2 | 6.4E-04 |
| 19 | Methyloceanibacter_superfactus | S1 | 20 | Dichotomicrobium_thermohalophilum | S1 | 0.82 | 2 | 1.1E-03 |
| 19 | Methyloceanibacter_superfactus | S1 | 11 | Nitrolancea_hollandica | S1 S2 | 0.73 | 1 | 6.8E-03 |
| 19 | Methyloceanibacter_superfactus | S1 | 21 | Patulibacter_medicamentivorans | S1 | 0.72 | 1 | 8.1E-03 |
| 19 | Methyloceanibacter_superfactus | S1 | 23 | Hydrogenibacillus_schlegelii | S1 S2 | 0.69 | 1 | 1.2E-02 |
| 20 | Dichotomicrobium_thermohalophilum | S1 | 21 | Patulibacter_medicamentivorans | S1 | 0.96 | 4 | 5.1E-07 |
| 20 | Dichotomicrobium_thermohalophilum | S1 | 18 | Thermoleophilum_album | S1 | 0.94 | 3 | 6.1E-06 |
| 20 | Dichotomicrobium_thermohalophilum | S1 | 16 | Singulisphaera_acidiphila | S1 S2 | 0.83 | 2 | 7.3E-04 |
| 20 | Dichotomicrobium_thermohalophilum | S1 | 17 | Limnochorda_pilosa | S1 S2 | 0.77 | 1 | 3.6E-03 |
| 20 | Dichotomicrobium_thermohalophilum | S1 | 22 | Bradyrhizobium_lablabi | S1 | 0.73 | 1 | 7.4E-03 |
| 20 | Dichotomicrobium_thermohalophilum | S1 | 12 | Paludisphaera_borealis | S1 S2 | 0.70 | 1 | 1.1E-02 |
| 20 | Dichotomicrobium_thermohalophilum | S1 | 14 | Kouleothrix_aurantiaca | S1 S2 | 0.67 | 1 | 1.7E-02 |
| 20 | Dichotomicrobium_thermohalophilum | S1 | 10 | Fimbriiglobus_ruber | S1 S2 | 0.61 | 1 | 3.6E-02 |
| 22 | Bradyrhizobium_lablabi | S1 | 21 | Patulibacter_medicamentivorans | S1 | 0.84 | 2 | 6.4E-04 |
| 22 | Bradyrhizobium_lablabi | S1 | 18 | Thermoleophilum_album | S1 | 0.66 | 1 | 2.0E-02 |
| 23 | Hydrogenibacillus_schlegelii | S1 S2 | 11 | Nitrolancea_hollandica | S1 S2 | 0.74 | 1 | 6.2E-03 |
| 23 | Hydrogenibacillus_schlegelii | S1 S2 | 14 | Kouleothrix_aurantiaca | S1 S2 | 0.72 | 1 | 8.2E-03 |
| 24 | Sutterella_parvirubra | S1 S2 | 11 | Nitrolancea_hollandica | S1 S2 | 0.92 | 3 | 2.9E-05 |
| 24 | Sutterella_parvirubra | S1 S2 | 10 | Fimbriiglobus_ruber | S1 S2 | 0.91 | 3 | 3.2E-05 |
| 24 | Sutterella_parvirubra | S1 S2 | 14 | Kouleothrix_aurantiaca | S1 S2 | 0.89 | 2 | 9.0E-05 |
| 24 | Sutterella_parvirubra | S1 S2 | 23 | Hydrogenibacillus_schlegelii | S1 S2 | 0.87 | 2 | 2.3E-04 |
| 24 | Sutterella_parvirubra | S1 S2 | 13 | Gemmata_obscuriglobus | S1 S2 | 0.86 | 2 | 3.9E-04 |
| 24 | Sutterella_parvirubra | S1 S2 | 12 | Paludisphaera_borealis | S1 S2 | 0.85 | 2 | 5.2E-04 |
| 24 | Sutterella_parvirubra | S1 S2 | 15 | Methyloceanibacter_marginalis | S1 S2 | 0.84 | 2 | 6.7E-04 |
| 24 | Sutterella_parvirubra | S1 S2 | 16 | Singulisphaera_acidiphila | S1 S2 | 0.79 | 1 | 2.5E-03 |
| 24 | Sutterella_parvirubra | S1 S2 | 19 | Methyloceanibacter_superfactus | S1 | 0.73 | 1 | 7.4E-03 |
| 24 | Sutterella_parvirubra | S1 S2 | 17 | Limnochorda_pilosa | S1 S2 | 0.71 | 1 | 1.0E-02 |
| 24 | Sutterella_parvirubra | S1 S2 | 18 | Thermoleophilum_album | S1 | 0.65 | 1 | 2.2E-02 |
| 25 | Leptonema_illini | S1SL S2SL | 33 | Stenotrophomonas_rhizophila | S1SL S2SL | 0.83 | 2 | 8.9E-04 |
| 25 | Leptonema_illini | S1SL S2SL | 29 | Thiobacillus_denitrificans | S1SL S2SL | 0.79 | 1 | 2.0E-03 |
| 25 | Leptonema_illini | S1SL S2SL | 35 | Thiobacillus_sp_65_29 | S1SL S2SL | 0.79 | 1 | 2.4E-03 |
| 25 | Leptonema_illini | S1SL S2SL | 30 | Lautropia_sp_SCN_69_89 | S1SL S2SL | 0.70 | 1 | 1.1E-02 |
| 25 | Leptonema_illini | S1SL S2SL | 32 | Ferrovibrio_sp | S1SL S2SL | 0.60 | 1 | 3.8E-02 |
| 25 | Leptonema_illini | S1SL S2SL | 31 | Rhodocyclales_bacterium | S1SL S2SL | 0.59 | 1 | 4.1E-02 |

**Table S4.** Continuation (3/6).

| **POSITIVE CORRELATIONS** | | | | | | | | |
| --- | --- | --- | --- | --- | --- | --- | --- | --- |
| **Base node** | | | **Target node** | | | **Edge parameters** | | |
| **no.** | **Taxa** | **Cluster** | **no.** | **Taxa** | **Cluster** | ***r_s_*** | **S** | ***p*** |
| 26 | Geobacter_anodireducens | S1SL S2SL | 35 | Thiobacillus_sp_65_29 | S1SL S2SL | 0.82 | 2 | 1.1E-03 |
| 26 | Geobacter_anodireducens | S1SL S2SL | 30 | Lautropia_sp_SCN_69_89 | S1SL S2SL | 0.73 | 1 | 7.3E-03 |
| 26 | Geobacter_anodireducens | S1SL S2SL | 38 | Fermentimonas_caenicola | S1SL S2SL | 0.65 | 1 | 2.4E-02 |
| 26 | Geobacter_anodireducens | S1SL S2SL | 32 | Ferrovibrio_sp | S1SL S2SL | 0.63 | 1 | 2.9E-02 |
| 26 | Geobacter_anodireducens | S1SL S2SL | 36 | Alicycliphilus_denitrificans | S1SL S2SL | 0.62 | 1 | 3.2E-02 |
| 27 | Geobacter_soli | S1SL S2SL | 26 | Geobacter_anodireducens | S1SL S2SL | 0.99 | 4 | 4.7E-11 |
| 27 | Geobacter_soli | S1SL S2SL | 35 | Thiobacillus_sp_65_29 | S1SL S2SL | 0.84 | 2 | 6.1E-04 |
| 27 | Geobacter_soli | S1SL S2SL | 30 | Lautropia_sp_SCN_69_89 | S1SL S2SL | 0.71 | 1 | 1.0E-02 |
| 27 | Geobacter_soli | S1SL S2SL | 38 | Fermentimonas_caenicola | S1SL S2SL | 0.65 | 1 | 2.3E-02 |
| 27 | Geobacter_soli | S1SL S2SL | 36 | Alicycliphilus_denitrificans | S1SL S2SL | 0.61 | 1 | 3.5E-02 |
| 27 | Geobacter_soli | S1SL S2SL | 32 | Ferrovibrio_sp | S1SL S2SL | 0.61 | 1 | 3.5E-02 |
| 28 | Geobacter_sulfurreducens | S1SL S2SL | 26 | Geobacter_anodireducens | S1SL S2SL | 1.00 | 4 | 2.7E-15 |
| 28 | Geobacter_sulfurreducens | S1SL S2SL | 27 | Geobacter_soli | S1SL S2SL | 0.99 | 4 | 1.3E-10 |
| 28 | Geobacter_sulfurreducens | S1SL S2SL | 35 | Thiobacillus_sp_65_29 | S1SL S2SL | 0.83 | 2 | 7.9E-04 |
| 28 | Geobacter_sulfurreducens | S1SL S2SL | 30 | Lautropia_sp_SCN_69_89 | S1SL S2SL | 0.73 | 1 | 6.8E-03 |
| 28 | Geobacter_sulfurreducens | S1SL S2SL | 32 | Ferrovibrio_sp | S1SL S2SL | 0.65 | 1 | 2.2E-02 |
| 28 | Geobacter_sulfurreducens | S1SL S2SL | 38 | Fermentimonas_caenicola | S1SL S2SL | 0.65 | 1 | 2.3E-02 |
| 28 | Geobacter_sulfurreducens | S1SL S2SL | 36 | Alicycliphilus_denitrificans | S1SL S2SL | 0.63 | 1 | 3.0E-02 |
| 29 | Thiobacillus_denitrificans | S1SL S2SL | 37 | Mycolicibacterium_fallax | S1SL S2SL | 0.69 | 1 | 1.3E-02 |
| 29 | Thiobacillus_denitrificans | S1SL S2SL | 36 | Alicycliphilus_denitrificans | S1SL S2SL | 0.67 | 1 | 1.7E-02 |
| 29 | Thiobacillus_denitrificans | S1SL S2SL | 8 | Sterolibacterium_denitrificans | SL | 0.66 | 1 | 1.9E-02 |
| 29 | Thiobacillus_denitrificans | S1SL S2SL | 6 | Parvibaculum_sp | SL | 0.64 | 1 | 2.6E-02 |
| 29 | Thiobacillus_denitrificans | S1SL S2SL | 7 | Methanosarcina_mazei | SL | 0.61 | 1 | 3.4E-02 |
| 29 | Thiobacillus_denitrificans | S1SL S2SL | 4 | Brachymonas_denitrificans | SL | 0.59 | 1 | 4.5E-02 |
| 30 | Lautropia_sp_SCN_69_89 | S1SL S2SL | 32 | Ferrovibrio_sp | S1SL S2SL | 0.64 | 1 | 2.5E-02 |
| 31 | Rhodocyclales_bacterium | S1SL S2SL | 30 | Lautropia_sp_SCN_69_89 | S1SL S2SL | 0.59 | 1 | 4.3E-02 |
| 33 | Stenotrophomonas_rhizophila | S1SL S2SL | 35 | Thiobacillus_sp_65_29 | S1SL S2SL | 0.89 | 2 | 1.0E-04 |
| 33 | Stenotrophomonas_rhizophila | S1SL S2SL | 27 | Geobacter_soli | S1SL S2SL | 0.88 | 2 | 1.8E-04 |
| 33 | Stenotrophomonas_rhizophila | S1SL S2SL | 26 | Geobacter_anodireducens | S1SL S2SL | 0.86 | 2 | 3.0E-04 |
| 33 | Stenotrophomonas_rhizophila | S1SL S2SL | 28 | Geobacter_sulfurreducens | S1SL S2SL | 0.86 | 2 | 3.0E-04 |
| 33 | Stenotrophomonas_rhizophila | S1SL S2SL | 30 | Lautropia_sp_SCN_69_89 | S1SL S2SL | 0.82 | 2 | 1.2E-03 |
| 33 | Stenotrophomonas_rhizophila | S1SL S2SL | 32 | Ferrovibrio_sp | S1SL S2SL | 0.65 | 1 | 2.1E-02 |
| 33 | Stenotrophomonas_rhizophila | S1SL S2SL | 29 | Thiobacillus_denitrificans | S1SL S2SL | 0.64 | 1 | 2.5E-02 |
| 33 | Stenotrophomonas_rhizophila | S1SL S2SL | 36 | Alicycliphilus_denitrificans | S1SL S2SL | 0.64 | 1 | 2.6E-02 |
| 33 | Stenotrophomonas_rhizophila | S1SL S2SL | 38 | Fermentimonas_caenicola | S1SL S2SL | 0.61 | 1 | 3.6E-02 |
| 34 | Nannocystis_exedens | S1SL S2SL | 23 | Hydrogenibacillus_schlegelii | S1 S2 | 0.68 | 1 | 1.4E-02 |
| 34 | Nannocystis_exedens | S1SL S2SL | 24 | Sutterella_parvirubra | S1 S2 | 0.67 | 1 | 1.7E-02 |
| 34 | Nannocystis_exedens | S1SL S2SL | 10 | Fimbriiglobus_ruber | S1 S2 | 0.63 | 1 | 2.9E-02 |
| 34 | Nannocystis_exedens | S1SL S2SL | 11 | Nitrolancea_hollandica | S1 S2 | 0.60 | 1 | 3.8E-02 |
| 34 | Nannocystis_exedens | S1SL S2SL | 13 | Gemmata_obscuriglobus | S1 S2 | 0.60 | 1 | 4.1E-02 |
| 34 | Nannocystis_exedens | S1SL S2SL | 14 | Kouleothrix_aurantiaca | S1 S2 | 0.59 | 1 | 4.4E-02 |
| 35 | Thiobacillus_sp_65_29 | S1SL S2SL | 32 | Ferrovibrio_sp | S1SL S2SL | 0.74 | 1 | 5.6E-03 |
| 35 | Thiobacillus_sp_65_29 | S1SL S2SL | 38 | Fermentimonas_caenicola | S1SL S2SL | 0.72 | 1 | 9.0E-03 |
| 35 | Thiobacillus_sp_65_29 | S1SL S2SL | 36 | Alicycliphilus_denitrificans | S1SL S2SL | 0.70 | 1 | 1.1E-02 |
| 35 | Thiobacillus_sp_65_29 | S1SL S2SL | 30 | Lautropia_sp_SCN_69_89 | S1SL S2SL | 0.68 | 1 | 1.6E-02 |
| 35 | Thiobacillus_sp_65_29 | S1SL S2SL | 29 | Thiobacillus_denitrificans | S1SL S2SL | 0.65 | 1 | 2.1E-02 |
| 36 | Alicycliphilus_denitrificans | S1SL S2SL | 38 | Fermentimonas_caenicola | S1SL S2SL | 0.79 | 1 | 2.1E-03 |
| 36 | Alicycliphilus_denitrificans | S1SL S2SL | 37 | Mycolicibacterium_fallax | S1SL S2SL | 0.72 | 1 | 8.7E-03 |
| 36 | Alicycliphilus_denitrificans | S1SL S2SL | 41 | Marinilabiliaceae_bacterium | S2SL | 0.70 | 1 | 1.0E-02 |
| 36 | Alicycliphilus_denitrificans | S1SL S2SL | 9 | Thermus_thermophilus | SL | 0.61 | 1 | 3.4E-02 |
| 36 | Alicycliphilus_denitrificans | S1SL S2SL | 6 | Parvibaculum_sp | SL | 0.59 | 1 | 4.2E-02 |
| 37 | Mycolicibacterium_fallax | S1SL S2SL | 7 | Methanosarcina_mazei | SL | 0.88 | 2 | 1.8E-04 |
| 38 | Fermentimonas_caenicola | S1SL S2SL | 40 | Bacteroidales_bacterium_6E | S2SL | 0.81 | 2 | 1.6E-03 |
| 39 | Acholeplasma_oculi | S2SL | 40 | Bacteroidales_bacterium_6E | S2SL | 0.97 | 4 | 2.2E-07 |
| 39 | Acholeplasma_oculi | S2SL | 41 | Marinilabiliaceae_bacterium | S2SL | 0.96 | 4 | 6.6E-07 |
| 39 | Acholeplasma_oculi | S2SL | 42 | Acholeplasma_laidlawii | S2SL | 0.87 | 2 | 2.4E-04 |
| 39 | Acholeplasma_oculi | S2SL | 38 | Fermentimonas_caenicola | S1SL S2SL | 0.86 | 2 | 3.8E-04 |
| 39 | Acholeplasma_oculi | S2SL | 43 | Acinetobacter_towneri | S2SL | 0.85 | 2 | 4.9E-04 |
| 39 | Acholeplasma_oculi | S2SL | 36 | Alicycliphilus_denitrificans | S1SL S2SL | 0.59 | 1 | 4.4E-02 |
| 41 | Marinilabiliaceae_bacterium | S2SL | 38 | Fermentimonas_caenicola | S1SL S2SL | 0.91 | 3 | 5.2E-05 |
| 41 | Marinilabiliaceae_bacterium | S2SL | 40 | Bacteroidales_bacterium_6E | S2SL | 0.89 | 2 | 1.0E-04 |
| 42 | Acholeplasma_laidlawii | S2SL | 43 | Acinetobacter_towneri | S2SL | 1.00 | 4 | 3.1E-13 |
| 42 | Acholeplasma_laidlawii | S2SL | 41 | Marinilabiliaceae_bacterium | S2SL | 0.89 | 2 | 8.5E-05 |
| 42 | Acholeplasma_laidlawii | S2SL | 9 | Thermus_thermophilus | SL | 0.79 | 1 | 2.4E-03 |
| 42 | Acholeplasma_laidlawii | S2SL | 40 | Bacteroidales_bacterium_6E | S2SL | 0.78 | 1 | 2.6E-03 |
| 42 | Acholeplasma_laidlawii | S2SL | 8 | Sterolibacterium_denitrificans | SL | 0.71 | 1 | 9.4E-03 |
| 42 | Acholeplasma_laidlawii | S2SL | 38 | Fermentimonas_caenicola | S1SL S2SL | 0.71 | 1 | 1.0E-02 |
| 42 | Acholeplasma_laidlawii | S2SL | 4 | Brachymonas_denitrificans | SL | 0.69 | 1 | 1.3E-02 |
| 42 | Acholeplasma_laidlawii | S2SL | 36 | Alicycliphilus_denitrificans | S1SL S2SL | 0.68 | 1 | 1.5E-02 |
| 42 | Acholeplasma_laidlawii | S2SL | 7 | Methanosarcina_mazei | SL | 0.65 | 1 | 2.3E-02 |
| 42 | Acholeplasma_laidlawii | S2SL | 2 | Stenotrophomonas_koreensis | SL | 0.62 | 1 | 3.0E-02 |

**Table S4.** Continuation (4/6).

| **POSITIVE CORRELATIONS** | | | | | | | | |
| --- | --- | --- | --- | --- | --- | --- | --- | --- |
| **Base node** | | | **Target node** | | | **Edge parameters** | | |
| **no.** | **Taxa** | **Cluster** | **no.** | **Taxa** | **Cluster** | ***r_s_*** | **S** | ***p*** |
| 42 | Acholeplasma_laidlawii | S2SL | 3 | Nitrosomonas_eutropha | SL | 0.61 | 1 | 3.5E-02 |
| 42 | Acholeplasma_laidlawii | S2SL | 1 | Comamonas_sp_SCN_65_56 | SL | 0.60 | 1 | 3.7E-02 |
| 42 | Acholeplasma_laidlawii | S2SL | 5 | Sphingopyxis_granuli | SL | 0.60 | 1 | 3.8E-02 |
| 43 | Acinetobacter_towneri | S2SL | 41 | Marinilabiliaceae_bacterium | S2SL | 0.88 | 2 | 1.3E-04 |
| 43 | Acinetobacter_towneri | S2SL | 9 | Thermus_thermophilus | SL | 0.81 | 2 | 1.3E-03 |
| 43 | Acinetobacter_towneri | S2SL | 40 | Bacteroidales_bacterium_6E | S2SL | 0.75 | 1 | 4.7E-03 |
| 43 | Acinetobacter_towneri | S2SL | 8 | Sterolibacterium_denitrificans | SL | 0.73 | 1 | 6.8E-03 |
| 43 | Acinetobacter_towneri | S2SL | 4 | Brachymonas_denitrificans | SL | 0.72 | 1 | 8.2E-03 |
| 43 | Acinetobacter_towneri | S2SL | 38 | Fermentimonas_caenicola | S1SL S2SL | 0.69 | 1 | 1.3E-02 |
| 43 | Acinetobacter_towneri | S2SL | 7 | Methanosarcina_mazei | SL | 0.68 | 1 | 1.5E-02 |
| 43 | Acinetobacter_towneri | S2SL | 36 | Alicycliphilus_denitrificans | S1SL S2SL | 0.67 | 1 | 1.7E-02 |
| 43 | Acinetobacter_towneri | S2SL | 3 | Nitrosomonas_eutropha | SL | 0.65 | 1 | 2.3E-02 |
| 43 | Acinetobacter_towneri | S2SL | 1 | Comamonas_sp_SCN_65_56 | SL | 0.64 | 1 | 2.5E-02 |
| 43 | Acinetobacter_towneri | S2SL | 5 | Sphingopyxis_granuli | SL | 0.64 | 1 | 2.6E-02 |
| 43 | Acinetobacter_towneri | S2SL | 6 | Parvibaculum_sp | SL | 0.60 | 1 | 3.8E-02 |

| **NEGATIVE CORRELATIONS** | | | | | | | | |
| --- | --- | --- | --- | --- | --- | --- | --- | --- |
| **Base node** | | | **Target node** | | | **Edge parameters** | | |
| **no.** | **Taxa** | **Cluster** | **no.** | **Taxa** | **Cluster** | ***r_s_*** | **S** | ***p*** |
| 8 | Sterolibacterium_denitrificans | SL | 14 | Kouleothrix_aurantiaca | S1 S2 | 0.58 | 1 | 4.9E-02 |
| 8 | Sterolibacterium_denitrificans | SL | 10 | Fimbriiglobus_ruber | S1 S2 | 0.59 | 1 | 4.3E-02 |
| 10 | Fimbriiglobus_ruber | S1 S2 | 41 | Marinilabiliaceae_bacterium | S2SL | 0.60 | 1 | 3.9E-02 |
| 10 | Fimbriiglobus_ruber | S1 S2 | 37 | Mycolicibacterium_fallax | S1SL S2SL | 0.63 | 1 | 2.9E-02 |
| 10 | Fimbriiglobus_ruber | S1 S2 | 38 | Fermentimonas_caenicola | S1SL S2SL | 0.77 | 1 | 3.5E-03 |
| 11 | Nitrolancea_hollandica | S1 S2 | 37 | Mycolicibacterium_fallax | S1SL S2SL | 0.58 | 1 | 4.7E-02 |
| 11 | Nitrolancea_hollandica | S1 S2 | 38 | Fermentimonas_caenicola | S1SL S2SL | 0.71 | 1 | 1.0E-02 |
| 12 | Paludisphaera_borealis | S1 S2 | 37 | Mycolicibacterium_fallax | S1SL S2SL | 0.58 | 1 | 4.6E-02 |
| 12 | Paludisphaera_borealis | S1 S2 | 38 | Fermentimonas_caenicola | S1SL S2SL | 0.75 | 1 | 5.4E-03 |
| 13 | Gemmata_obscuriglobus | S1 S2 | 37 | Mycolicibacterium_fallax | S1SL S2SL | 0.61 | 1 | 3.4E-02 |
| 13 | Gemmata_obscuriglobus | S1 S2 | 38 | Fermentimonas_caenicola | S1SL S2SL | 0.68 | 1 | 1.5E-02 |
| 14 | Kouleothrix_aurantiaca | S1 S2 | 41 | Marinilabiliaceae_bacterium | S2SL | 0.60 | 1 | 3.8E-02 |
| 14 | Kouleothrix_aurantiaca | S1 S2 | 37 | Mycolicibacterium_fallax | S1SL S2SL | 0.61 | 1 | 3.4E-02 |
| 14 | Kouleothrix_aurantiaca | S1 S2 | 38 | Fermentimonas_caenicola | S1SL S2SL | 0.78 | 1 | 2.5E-03 |
| 15 | Methyloceanibacter_marginalis | S1 S2 | 41 | Marinilabiliaceae_bacterium | S2SL | 0.59 | 1 | 4.5E-02 |
| 15 | Methyloceanibacter_marginalis | S1 S2 | 37 | Mycolicibacterium_fallax | S1SL S2SL | 0.60 | 1 | 4.0E-02 |
| 15 | Methyloceanibacter_marginalis | S1 S2 | 38 | Fermentimonas_caenicola | S1SL S2SL | 0.76 | 1 | 3.8E-03 |
| 16 | Singulisphaera_acidiphila | S1 S2 | 41 | Marinilabiliaceae_bacterium | S2SL | 0.60 | 1 | 3.9E-02 |
| 16 | Singulisphaera_acidiphila | S1 S2 | 37 | Mycolicibacterium_fallax | S1SL S2SL | 0.60 | 1 | 3.8E-02 |
| 16 | Singulisphaera_acidiphila | S1 S2 | 38 | Fermentimonas_caenicola | S1SL S2SL | 0.78 | 1 | 2.9E-03 |
| 17 | Limnochorda_pilosa | S1 S2 | 38 | Fermentimonas_caenicola | S1SL S2SL | 0.73 | 1 | 7.4E-03 |
| 18 | Thermoleophilum_album | S1 | 37 | Mycolicibacterium_fallax | S1SL S2SL | 0.58 | 1 | 4.8E-02 |
| 19 | Methyloceanibacter_superfactus | S1 | 38 | Fermentimonas_caenicola | S1SL S2SL | 0.73 | 1 | 7.1E-03 |
| 20 | Dichotomicrobium_thermohalophilum | S1 | 38 | Fermentimonas_caenicola | S1SL S2SL | 0.64 | 1 | 2.4E-02 |
| 24 | Sutterella_parvirubra | S1 S2 | 38 | Fermentimonas_caenicola | S1SL S2SL | 0.67 | 1 | 1.7E-02 |
| 24 | Sutterella_parvirubra | S1 S2 | 36 | Alicycliphilus_denitrificans | S1SL S2SL | 0.78 | 1 | 2.5E-03 |
| 25 | Leptonema_illini | S1SL S2SL | 24 | Sutterella_parvirubra | S1 S2 | 0.64 | 1 | 2.4E-02 |
| 25 | Leptonema_illini | S1SL S2SL | 18 | Thermoleophilum_album | S1 | 0.70 | 1 | 1.2E-02 |
| 25 | Leptonema_illini | S1SL S2SL | 11 | Nitrolancea_hollandica | S1 S2 | 0.70 | 1 | 1.2E-02 |
| 25 | Leptonema_illini | S1SL S2SL | 14 | Kouleothrix_aurantiaca | S1 S2 | 0.70 | 1 | 1.1E-02 |
| 25 | Leptonema_illini | S1SL S2SL | 19 | Methyloceanibacter_superfactus | S1 | 0.70 | 1 | 1.1E-02 |
| 25 | Leptonema_illini | S1SL S2SL | 12 | Paludisphaera_borealis | S1 S2 | 0.71 | 1 | 1.0E-02 |
| 25 | Leptonema_illini | S1SL S2SL | 10 | Fimbriiglobus_ruber | S1 S2 | 0.71 | 1 | 1.0E-02 |
| 25 | Leptonema_illini | S1SL S2SL | 13 | Gemmata_obscuriglobus | S1 S2 | 0.71 | 1 | 9.0E-03 |
| 25 | Leptonema_illini | S1SL S2SL | 17 | Limnochorda_pilosa | S1 S2 | 0.72 | 1 | 8.7E-03 |
| 25 | Leptonema_illini | S1SL S2SL | 15 | Methyloceanibacter_marginalis | S1 S2 | 0.74 | 1 | 6.4E-03 |
| 25 | Leptonema_illini | S1SL S2SL | 16 | Singulisphaera_acidiphila | S1 S2 | 0.74 | 1 | 6.2E-03 |
| 26 | Geobacter_anodireducens | S1SL S2SL | 23 | Hydrogenibacillus_schlegelii | S1 S2 | 0.60 | 1 | 3.9E-02 |
| 26 | Geobacter_anodireducens | S1SL S2SL | 20 | Dichotomicrobium_thermohalophilum | S1 | 0.67 | 1 | 1.8E-02 |
| 26 | Geobacter_anodireducens | S1SL S2SL | 18 | Thermoleophilum_album | S1 | 0.69 | 1 | 1.3E-02 |
| 26 | Geobacter_anodireducens | S1SL S2SL | 11 | Nitrolancea_hollandica | S1 S2 | 0.70 | 1 | 1.2E-02 |
| 26 | Geobacter_anodireducens | S1SL S2SL | 19 | Methyloceanibacter_superfactus | S1 | 0.71 | 1 | 9.8E-03 |
| 26 | Geobacter_anodireducens | S1SL S2SL | 13 | Gemmata_obscuriglobus | S1 S2 | 0.71 | 1 | 9.6E-03 |
| 26 | Geobacter_anodireducens | S1SL S2SL | 24 | Sutterella_parvirubra | S1 S2 | 0.73 | 1 | 7.4E-03 |
| 26 | Geobacter_anodireducens | S1SL S2SL | 10 | Fimbriiglobus_ruber | S1 S2 | 0.74 | 1 | 6.3E-03 |
| 26 | Geobacter_anodireducens | S1SL S2SL | 17 | Limnochorda_pilosa | S1 S2 | 0.75 | 1 | 4.8E-03 |
| 26 | Geobacter_anodireducens | S1SL S2SL | 15 | Methyloceanibacter_marginalis | S1 S2 | 0.76 | 1 | 4.2E-03 |
| 26 | Geobacter_anodireducens | S1SL S2SL | 16 | Singulisphaera_acidiphila | S1 S2 | 0.76 | 1 | 4.1E-03 |
| 26 | Geobacter_anodireducens | S1SL S2SL | 14 | Kouleothrix_aurantiaca | S1 S2 | 0.77 | 1 | 3.6E-03 |
| 26 | Geobacter_anodireducens | S1SL S2SL | 12 | Paludisphaera_borealis | S1 S2 | 0.78 | 1 | 2.9E-03 |

**Table S4.** Continuation (5/6).

| **NEGATIVE CORRELATIONS** | | | | | | | | |
| --- | --- | --- | --- | --- | --- | --- | --- | --- |
| **Base node** | | | **Target node** | | | **Edge parameters** | | |
| **no.** | **Taxa** | **Cluster** | **no.** | **Taxa** | **Cluster** | ***r_s_*** | **S** | ***p*** |
| 27 | Geobacter_soli | S1SL S2SL | 23 | Hydrogenibacillus_schlegelii | S1 S2 | 0.61 | 1 | 3.6E-02 |
| 27 | Geobacter_soli | S1SL S2SL | 20 | Dichotomicrobium_thermohalophilum | S1 | 0.68 | 1 | 1.5E-02 |
| 27 | Geobacter_soli | S1SL S2SL | 18 | Thermoleophilum_album | S1 | 0.71 | 1 | 9.6E-03 |
| 27 | Geobacter_soli | S1SL S2SL | 11 | Nitrolancea_hollandica | S1 S2 | 0.71 | 1 | 9.4E-03 |
| 27 | Geobacter_soli | S1SL S2SL | 13 | Gemmata_obscuriglobus | S1 S2 | 0.72 | 1 | 7.8E-03 |
| 27 | Geobacter_soli | S1SL S2SL | 19 | Methyloceanibacter_superfactus | S1 | 0.73 | 1 | 7.3E-03 |
| 27 | Geobacter_soli | S1SL S2SL | 24 | Sutterella_parvirubra | S1 S2 | 0.74 | 1 | 5.6E-03 |
| 27 | Geobacter_soli | S1SL S2SL | 10 | Fimbriiglobus_ruber | S1 S2 | 0.75 | 1 | 5.1E-03 |
| 27 | Geobacter_soli | S1SL S2SL | 17 | Limnochorda_pilosa | S1 S2 | 0.77 | 1 | 3.5E-03 |
| 27 | Geobacter_soli | S1SL S2SL | 16 | Singulisphaera_acidiphila | S1 S2 | 0.77 | 1 | 3.1E-03 |
| 27 | Geobacter_soli | S1SL S2SL | 15 | Methyloceanibacter_marginalis | S1 S2 | 0.78 | 1 | 2.9E-03 |
| 27 | Geobacter_soli | S1SL S2SL | 14 | Kouleothrix_aurantiaca | S1 S2 | 0.78 | 1 | 2.9E-03 |
| 27 | Geobacter_soli | S1SL S2SL | 12 | Paludisphaera_borealis | S1 S2 | 0.79 | 1 | 2.2E-03 |
| 28 | Geobacter_sulfurreducens | S1SL S2SL | 23 | Hydrogenibacillus_schlegelii | S1 S2 | 0.60 | 1 | 3.8E-02 |
| 28 | Geobacter_sulfurreducens | S1SL S2SL | 20 | Dichotomicrobium_thermohalophilum | S1 | 0.67 | 1 | 1.8E-02 |
| 28 | Geobacter_sulfurreducens | S1SL S2SL | 18 | Thermoleophilum_album | S1 | 0.69 | 1 | 1.3E-02 |
| 28 | Geobacter_sulfurreducens | S1SL S2SL | 11 | Nitrolancea_hollandica | S1 S2 | 0.70 | 1 | 1.2E-02 |
| 28 | Geobacter_sulfurreducens | S1SL S2SL | 19 | Methyloceanibacter_superfactus | S1 | 0.71 | 1 | 9.6E-03 |
| 28 | Geobacter_sulfurreducens | S1SL S2SL | 13 | Gemmata_obscuriglobus | S1 S2 | 0.71 | 1 | 9.2E-03 |
| 28 | Geobacter_sulfurreducens | S1SL S2SL | 24 | Sutterella_parvirubra | S1 S2 | 0.73 | 1 | 6.9E-03 |
| 28 | Geobacter_sulfurreducens | S1SL S2SL | 10 | Fimbriiglobus_ruber | S1 S2 | 0.74 | 1 | 6.0E-03 |
| 28 | Geobacter_sulfurreducens | S1SL S2SL | 17 | Limnochorda_pilosa | S1 S2 | 0.75 | 1 | 4.7E-03 |
| 28 | Geobacter_sulfurreducens | S1SL S2SL | 15 | Methyloceanibacter_marginalis | S1 S2 | 0.76 | 1 | 4.1E-03 |
| 28 | Geobacter_sulfurreducens | S1SL S2SL | 16 | Singulisphaera_acidiphila | S1 S2 | 0.77 | 1 | 3.7E-03 |
| 28 | Geobacter_sulfurreducens | S1SL S2SL | 14 | Kouleothrix_aurantiaca | S1 S2 | 0.77 | 1 | 3.4E-03 |
| 28 | Geobacter_sulfurreducens | S1SL S2SL | 12 | Paludisphaera_borealis | S1 S2 | 0.78 | 1 | 2.7E-03 |
| 29 | Thiobacillus_denitrificans | S1SL S2SL | 21 | Patulibacter_medicamentivorans | S1 | 0.60 | 1 | 4.1E-02 |
| 29 | Thiobacillus_denitrificans | S1SL S2SL | 24 | Sutterella_parvirubra | S1 S2 | 0.66 | 1 | 2.0E-02 |
| 29 | Thiobacillus_denitrificans | S1SL S2SL | 19 | Methyloceanibacter_superfactus | S1 | 0.75 | 1 | 5.0E-03 |
| 29 | Thiobacillus_denitrificans | S1SL S2SL | 11 | Nitrolancea_hollandica | S1 S2 | 0.75 | 1 | 4.8E-03 |
| 29 | Thiobacillus_denitrificans | S1SL S2SL | 12 | Paludisphaera_borealis | S1 S2 | 0.75 | 1 | 4.7E-03 |
| 29 | Thiobacillus_denitrificans | S1SL S2SL | 17 | Limnochorda_pilosa | S1 S2 | 0.75 | 1 | 4.6E-03 |
| 29 | Thiobacillus_denitrificans | S1SL S2SL | 18 | Thermoleophilum_album | S1 | 0.76 | 1 | 4.2E-03 |
| 29 | Thiobacillus_denitrificans | S1SL S2SL | 14 | Kouleothrix_aurantiaca | S1 S2 | 0.78 | 1 | 2.9E-03 |
| 29 | Thiobacillus_denitrificans | S1SL S2SL | 13 | Gemmata_obscuriglobus | S1 S2 | 0.78 | 1 | 2.8E-03 |
| 29 | Thiobacillus_denitrificans | S1SL S2SL | 15 | Methyloceanibacter_marginalis | S1 S2 | 0.78 | 1 | 2.5E-03 |
| 29 | Thiobacillus_denitrificans | S1SL S2SL | 10 | Fimbriiglobus_ruber | S1 S2 | 0.80 | 1 | 2.0E-03 |
| 29 | Thiobacillus_denitrificans | S1SL S2SL | 16 | Singulisphaera_acidiphila | S1 S2 | 0.80 | 1 | 1.8E-03 |
| 30 | Lautropia_sp_SCN_69_89 | S1SL S2SL | 18 | Thermoleophilum_album | S1 | 0.59 | 1 | 4.6E-02 |
| 30 | Lautropia_sp_SCN_69_89 | S1SL S2SL | 11 | Nitrolancea_hollandica | S1 S2 | 0.59 | 1 | 4.1E-02 |
| 30 | Lautropia_sp_SCN_69_89 | S1SL S2SL | 19 | Methyloceanibacter_superfactus | S1 | 0.61 | 1 | 3.7E-02 |
| 30 | Lautropia_sp_SCN_69_89 | S1SL S2SL | 10 | Fimbriiglobus_ruber | S1 S2 | 0.63 | 1 | 2.8E-02 |
| 30 | Lautropia_sp_SCN_69_89 | S1SL S2SL | 15 | Methyloceanibacter_marginalis | S1 S2 | 0.63 | 1 | 2.7E-02 |
| 30 | Lautropia_sp_SCN_69_89 | S1SL S2SL | 12 | Paludisphaera_borealis | S1 S2 | 0.64 | 1 | 2.6E-02 |
| 30 | Lautropia_sp_SCN_69_89 | S1SL S2SL | 14 | Kouleothrix_aurantiaca | S1 S2 | 0.64 | 1 | 2.5E-02 |
| 30 | Lautropia_sp_SCN_69_89 | S1SL S2SL | 13 | Gemmata_obscuriglobus | S1 S2 | 0.64 | 1 | 2.5E-02 |
| 30 | Lautropia_sp_SCN_69_89 | S1SL S2SL | 16 | Singulisphaera_acidiphila | S1 S2 | 0.65 | 1 | 2.3E-02 |
| 30 | Lautropia_sp_SCN_69_89 | S1SL S2SL | 17 | Limnochorda_pilosa | S1 S2 | 0.65 | 1 | 2.2E-02 |
| 33 | Stenotrophomonas_rhizophila | S1SL S2SL | 23 | Hydrogenibacillus_schlegelii | S1 S2 | 0.58 | 1 | 4.7E-02 |
| 33 | Stenotrophomonas_rhizophila | S1SL S2SL | 21 | Patulibacter_medicamentivorans | S1 | 0.71 | 1 | 9.3E-03 |
| 33 | Stenotrophomonas_rhizophila | S1SL S2SL | 24 | Sutterella_parvirubra | S1 S2 | 0.73 | 1 | 7.3E-03 |
| 33 | Stenotrophomonas_rhizophila | S1SL S2SL | 11 | Nitrolancea_hollandica | S1 S2 | 0.75 | 1 | 4.9E-03 |
| 33 | Stenotrophomonas_rhizophila | S1SL S2SL | 20 | Dichotomicrobium_thermohalophilum | S1 | 0.81 | 2 | 1.5E-03 |
| 33 | Stenotrophomonas_rhizophila | S1SL S2SL | 10 | Fimbriiglobus_ruber | S1 S2 | 0.82 | 2 | 1.2E-03 |
| 33 | Stenotrophomonas_rhizophila | S1SL S2SL | 13 | Gemmata_obscuriglobus | S1 S2 | 0.83 | 2 | 8.3E-04 |
| 33 | Stenotrophomonas_rhizophila | S1SL S2SL | 14 | Kouleothrix_aurantiaca | S1 S2 | 0.83 | 2 | 7.3E-04 |
| 33 | Stenotrophomonas_rhizophila | S1SL S2SL | 17 | Limnochorda_pilosa | S1 S2 | 0.86 | 2 | 3.4E-04 |
| 33 | Stenotrophomonas_rhizophila | S1SL S2SL | 19 | Methyloceanibacter_superfactus | S1 | 0.86 | 2 | 3.3E-04 |
| 33 | Stenotrophomonas_rhizophila | S1SL S2SL | 18 | Thermoleophilum_album | S1 | 0.86 | 2 | 3.1E-04 |
| 33 | Stenotrophomonas_rhizophila | S1SL S2SL | 12 | Paludisphaera_borealis | S1 S2 | 0.89 | 2 | 9.6E-05 |
| 33 | Stenotrophomonas_rhizophila | S1SL S2SL | 15 | Methyloceanibacter_marginalis | S1 S2 | 0.90 | 2 | 6.8E-05 |
| 33 | Stenotrophomonas_rhizophila | S1SL S2SL | 16 | Singulisphaera_acidiphila | S1 S2 | 0.90 | 3 | 6.3E-05 |
| 34 | Nannocystis_exedens | S1SL S2SL | 37 | Mycolicibacterium_fallax | S1SL S2SL | 0.60 | 1 | 4.0E-02 |
| 34 | Nannocystis_exedens | S1SL S2SL | 8 | Sterolibacterium_denitrificans | SL | 0.69 | 1 | 1.2E-02 |
| 34 | Nannocystis_exedens | S1SL S2SL | 36 | Alicycliphilus_denitrificans | S1SL S2SL | 0.74 | 1 | 6.4E-03 |
| 35 | Thiobacillus_sp_65_29 | S1SL S2SL | 21 | Patulibacter_medicamentivorans | S1 | 0.62 | 1 | 3.1E-02 |
| 35 | Thiobacillus_sp_65_29 | S1SL S2SL | 23 | Hydrogenibacillus_schlegelii | S1 S2 | 0.66 | 1 | 1.9E-02 |
| 35 | Thiobacillus_sp_65_29 | S1SL S2SL | 20 | Dichotomicrobium_thermohalophilum | S1 | 0.71 | 1 | 9.1E-03 |
| 35 | Thiobacillus_sp_65_29 | S1SL S2SL | 18 | Thermoleophilum_album | S1 | 0.80 | 1 | 1.9E-03 |
| 35 | Thiobacillus_sp_65_29 | S1SL S2SL | 11 | Nitrolancea_hollandica | S1 S2 | 0.81 | 2 | 1.6E-03 |
| 35 | Thiobacillus_sp_65_29 | S1SL S2SL | 13 | Gemmata_obscuriglobus | S1 S2 | 0.81 | 2 | 1.5E-03 |

**Table S4.** Continuation (6/6).

| **NEGATIVE CORRELATIONS** | | | | | | | | |
| --- | --- | --- | --- | --- | --- | --- | --- | --- |
| **Base node** | | | **Target node** | | | **Edge parameters** | | |
| **no.** | **Taxa** | **Cluster** | **no.** | **Taxa** | **Cluster** | ***r_s_*** | **S** | ***p*** |
| 35 | Thiobacillus_sp_65_29 | S1SL S2SL | 24 | Sutterella_parvirubra | S1 S2 | 0.82 | 2 | 1.2E-03 |
| 35 | Thiobacillus_sp_65_29 | S1SL S2SL | 19 | Methyloceanibacter_superfactus | S1 | 0.82 | 2 | 1.2E-03 |
| 35 | Thiobacillus_sp_65_29 | S1SL S2SL | 17 | Limnochorda_pilosa | S1 S2 | 0.84 | 2 | 7.1E-04 |
| 35 | Thiobacillus_sp_65_29 | S1SL S2SL | 10 | Fimbriiglobus_ruber | S1 S2 | 0.85 | 2 | 5.0E-04 |
| 35 | Thiobacillus_sp_65_29 | S1SL S2SL | 15 | Methyloceanibacter_marginalis | S1 S2 | 0.86 | 2 | 3.1E-04 |
| 35 | Thiobacillus_sp_65_29 | S1SL S2SL | 12 | Paludisphaera_borealis | S1 S2 | 0.86 | 2 | 3.0E-04 |
| 35 | Thiobacillus_sp_65_29 | S1SL S2SL | 14 | Kouleothrix_aurantiaca | S1 S2 | 0.86 | 2 | 2.9E-04 |
| 35 | Thiobacillus_sp_65_29 | S1SL S2SL | 16 | Singulisphaera_acidiphila | S1 S2 | 0.89 | 2 | 1.3E-04 |
| 36 | Alicycliphilus_denitrificans | S1SL S2SL | 21 | Patulibacter_medicamentivorans | S1 | 0.60 | 1 | 3.9E-02 |
| 36 | Alicycliphilus_denitrificans | S1SL S2SL | 23 | Hydrogenibacillus_schlegelii | S1 S2 | 0.62 | 1 | 3.3E-02 |
| 36 | Alicycliphilus_denitrificans | S1SL S2SL | 20 | Dichotomicrobium_thermohalophilum | S1 | 0.63 | 1 | 2.8E-02 |
| 36 | Alicycliphilus_denitrificans | S1SL S2SL | 17 | Limnochorda_pilosa | S1 S2 | 0.79 | 1 | 2.3E-03 |
| 36 | Alicycliphilus_denitrificans | S1SL S2SL | 18 | Thermoleophilum_album | S1 | 0.79 | 1 | 2.3E-03 |
| 36 | Alicycliphilus_denitrificans | S1SL S2SL | 19 | Methyloceanibacter_superfactus | S1 | 0.79 | 1 | 2.1E-03 |
| 36 | Alicycliphilus_denitrificans | S1SL S2SL | 11 | Nitrolancea_hollandica | S1 S2 | 0.80 | 1 | 1.9E-03 |
| 36 | Alicycliphilus_denitrificans | S1SL S2SL | 13 | Gemmata_obscuriglobus | S1 S2 | 0.80 | 2 | 1.8E-03 |
| 36 | Alicycliphilus_denitrificans | S1SL S2SL | 15 | Methyloceanibacter_marginalis | S1 S2 | 0.84 | 2 | 6.2E-04 |
| 36 | Alicycliphilus_denitrificans | S1SL S2SL | 12 | Paludisphaera_borealis | S1 S2 | 0.85 | 2 | 5.2E-04 |
| 36 | Alicycliphilus_denitrificans | S1SL S2SL | 10 | Fimbriiglobus_ruber | S1 S2 | 0.87 | 2 | 2.5E-04 |
| 36 | Alicycliphilus_denitrificans | S1SL S2SL | 16 | Singulisphaera_acidiphila | S1 S2 | 0.87 | 2 | 2.3E-04 |
| 36 | Alicycliphilus_denitrificans | S1SL S2SL | 14 | Kouleothrix_aurantiaca | S1 S2 | 0.88 | 2 | 1.8E-04 |
| 38 | Fermentimonas_caenicola | S1SL S2SL | 18 | Thermoleophilum_album | S1 | 0.72 | 1 | 8.3E-03 |

Base nodes represent the taxa (prokaryote species) that contained the higher number of significant co-occurrences with other taxa (represented here as the target nodes). *r_s_* = Spearman’s correlation. Only significant co-occurrences were added (*p* < 0.05). The correlation, whether its positive or negative, was categorized according to the strength of the Spearman’s value: (4) *r_s_* ≥ 0.96, (3) 0.95 ≤ *r_s_* ≥ 0.90, (2) 0.89 ≤ *r_s_* ≥ 0.80, and (1) 0.79 ≤ *r_s_* ≥ 0.56.

**Table S5**. Summary of significant (*p* < 0.05) positive and negative Spearman correlations (*r_s_*) between the different clusters identified for the network analysis.

The strength of the correlation was stratified according to the Spearman’s value: (4) *r_s_* ≥ 0.96, (3) 0.95 ≤ *r_s_* ≥ 0.90, (2) 0.89 ≤ *r_s_* ≥ 0.80, and (1) 0.79 ≤ *r_s_* ≥ 0.56.

**Table S6.** PCoA scores for soilt treatments

| Sample - replicate | Coordinate 1 (72.6%) | Coordinate 2 (12.9%) |
| --- | --- | --- |
| S2 -1 | 0.33067 | 0.030149 |
| S2 -2 | 0.35649 | 0.053088 |
| S2 -3 | 0.32819 | 0.030619 |
| S2SL -1 | -0.23826 | -0.44083 |
| S2SL -2 | -0.26463 | -0.030936 |
| S2SL -3 | -0.17988 | 0.29027 |
| S1 -1 | 0.34661 | 0.058188 |
| S1 -2 | 0.30913 | 0.032515 |
| S1SL -1 | -0.29604 | 0.005951 |
| S1SL -2 | -0.31145 | 0.5965 |
| S1SL -3 | -0.2996 | -0.03004 |
| SL | -0.08124 | -0.59548 |

Scores highlighted in blue were utilized for statistical analysis described in Table S7.

**Table S7.** Statistical non-parametric Kruskal Wallis (a) and Mann-Whitney post-hoc (b) analyses to test significant differences between soil treatments according to the prokaryotic composition.

1. Kruskal Wallis test

| Parameter | Samples (n = 11) |
| --- | --- |
| H (chi^2^): | 9.667 |
| Hc (tie corrected): | 9.667 |
| p (same): | 0.02162 |

Conclusion: There is a significant difference between sample medians (highlighted in red colour), Mann-Whitney post-hoc test was performed to observe specific differences between soil treatments.

1. Mann-Whitney post-hot test

| Soil treatments | S1 | S2 | S2SL | S1SL |
| --- | --- | --- | --- | --- |
| S1 |  | 0.9534 | 1.81E-08 | 9.80E-09 |
| S2 | 0.7268 |  | 1.65E-08 | 8.99E-09 |
| S2SL | 38.16 | 38.88 |  | 0.02747 |
| S1SL | 43.29 | 44.02 | 5.137 |  |

Sewage sludge (SL) was not considered for the present statistical analysis due to only one replicate.

**Table S8.** Number of oligotrophic and copiotrophic bacteria before and 50 days after sewage sludge was added.

| **Sample** | **day 0** | | |  | **day 50** | | |
| --- | --- | --- | --- | --- | --- | --- | --- |
|  | Oligotrophs | Copiotrophs | ratio (O:C) |  | Oligotrophs | Copiotrophs | ratio (O:C) |
| SL | 1,45E+09 ± 0.70 | 4,9E+09 ± 0.71 | 0.3:1 |  | 1,3E+09 ± 0.64 | 5,0E+09 ± 0.75 | ~ 0.3:1 |
| S1 | 7,8E+06 ± 0.16 | 3,9E+06 ± 0.17 | 02:01 |  | 7,7E+06 ± 0.21 | 4,0E+06 ± 0.15 | ~ 2:1 |
| S1SL | 9,8E+07 ± 0.39 | 4,7E+07 ± 0.31 | 02:01 |  | 5,2E+07 ± 0.40 | 4,5E+07 ± 0.38 | ~ 1:1 |
| S2 | 7,2E+07 ± 0.14 | 2,1E+07 ± 0.23 | 3.4:1 |  | 7,0E+07 ± 0.11 | 2,0E+07 ± 0.19 | 3.5:1 |
| S2SL | 2,3E+08 ± 0.37 | 3,0E+08 ± 0.37 | 0.76:1 |  | 1,2E+08 ± 0.36 | 3,5E+08 ± 0.41 | 0.34:1 |

Ratio is presented as the value of oligotrophs over the value of copiotrophs (O:C).

**
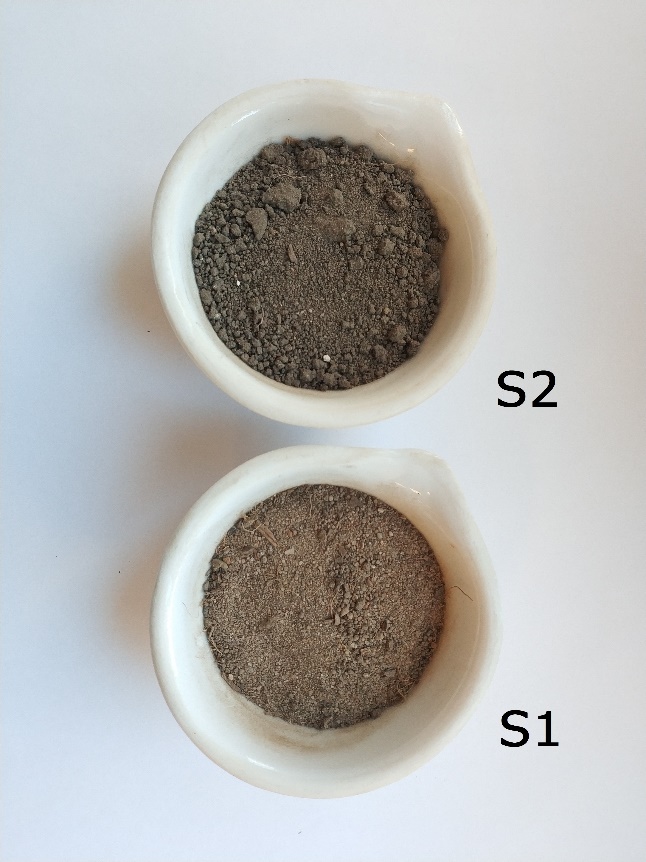
**

**Fig S2.** Pictures of control soils S1 and S2.
